# Cardiopulmonary bypass and internal thoracic artery: Can roller or centrifugal pumps change vascular reactivity of the graft? The IPITA study: A randomized controlled clinical trial

**DOI:** 10.1371/journal.pone.0235604

**Published:** 2020-07-09

**Authors:** Olivier Fouquet, Simon Dang Van, Anna Baudry, Philippe Meisnerowski, Pauline Robert, Frédéric Pinaud, Patrice Binuani, Jean-Marie Chrétien, Daniel Henrion, Christophe Baufreton, Laurent Loufrani

**Affiliations:** 1 Department of Thoracic and Cardiovascular Surgery, University Hospital, Angers, France; 2 MITOVASC Institute CNRS UMR 6214, INSERM U1083, University of Angers, Angers, France; 3 Clinical Research Department, University Hospital, Angers, France; Universita degli Studi Magna Graecia di Catanzaro, ITALY

## Abstract

**Background:**

Cardiopulmonary bypass (CPB) induces a systemic inflammatory response (SIRS) and affects the organ vascular bed. Experimentally, the lack of pulsatility alters myogenic tone of resistance arteries and increases the parietal inflammatory response. The purpose of this study was to compare the vascular reactivity of the internal thoracic arteries (ITAs) due to the inflammatory response between patients undergoing coronary artery bypass grafting (CABG) under CPB with a roller pump or with a centrifugal pump.

**Methods:**

Eighty elective male patients undergoing CABG were selected using one or two internal thoracic arteries under CPB with a roller pump (RP group) or centrifugal pump (CFP group). ITA samples were collected before starting CPB (Time 1) and before the last coronary anastomosis during aortic cross clamping (Time 2). The primary endpoint was the endothelium-dependent relaxation of ITAs investigated using wire-myography. The secondary endpoint was the parietal inflammatory response of arteries defined by the measurements of superoxide levels, leukocytes and lymphocytes rate and gene expression of inflammatory proteins using. Terminal complement complex activation (SC5b-9) and neutrophil activation (elastase) analysis were performed on arterial blood at the same times.

**Results:**

Exposure time of ITAs to the pump flow was respectively 43.3 minutes in the RP group and 45.7 minutes in the CFP group. Acetylcholine-dependent relaxation was conserved in the two groups whatever the time. Gene expression of C3 and C4a in the artery wall decreased from Time 1 to Time 2. No oxidative stress was observed in the graft. There was no difference between the groups concerning the leukocytes and lymphocytes rate. SC5b-9 and elastase increased between Time 1 and Time 2.

**Conclusion:**

Endothelium-dependent relaxation of the internal thoracic arteries was preserved during CPB whatever the type of pump used. The inflammatory response observed in the blood was not found in the graft wall within this time frame.

**Trial registration:**

Name of trial study protocol: IPITA

Registration number (ClinicalTrials.gov): NCT04168853.

## Introduction

More than 12000 coronary artery bypass grafting (CABG) procedures under cardiopulmonary bypass (CPB) are performed each year in France, 60% using a roller pump (RP) and 40% using a centrifugal pump (CFP) [1].

The superiority of the CFP over the RP is debated. The CFP is considered safer than the RP by reducing the risk of microgaseous emboli and hemolysis [2]. However, a recent meta-analysis [3] failed to demonstrate any clinical difference between the two types of arterial pumps used for CPB, but this comparison was limited to clinical events and did not focus on the systemic inflammatory response syndrome (SIRS). Although the CFP was expected to reduce the SIRS, initial comparisons between the two pumps have shown that the RP, when compared to CFP, decreased the terminal complement complex activation (SC5b-9) as well as elastase release during coronary artery bypass grafting (CABG) under CPB [4, 5]. It has long been reported that the RP produces a pulsatile flow, which is responsible for lateral transmission of energy to tissue, whereas the CFP produces a non-pulsatile flow without an add-on device [6, 7]. The lack of pulsatile flow may increase some aspects of SIRS such as endotoxin release during aortic cross clamping in patients undergoing CABG [8]. We previously demonstrated experimentally that an inflammatory reaction was less damaging for the mesenteric arteries of rats subjected to very long pulsatile perfusion than arteries submitted to non-pulsatile perfusion, independently of flow or pressure [9]. In addition, these effects originated directly within the arterial wall and not from a blood activation process, since these mesenteric arteries were perfused with a bloodless saline solution. Interestingly, patients undergoing non pulsatile mechanical circulatory support are prone to gastrointestinal injury and bleeding [10], an effect that is usually related to a shear stress mediated Willebrand factor defect [11] rather than to a local organic inflammatory process. Nevertheless, impaired vasorelaxation and propensity to vasospasm caused by SIRS during CPB may affect several vascular territories such as the pulmonary, mesenteric or cerebral microvascularization [12]. By modifying the SIRS using heparin-coated circuits, we observed a significant reduction of vasospasm in the cerebral arteries using transcranial Doppler ultrasound in patients undergoing CABG [13]. Therefore, we postulated that such a detectable Doppler benefit of SIRS reduction might also be observed in the internal thoracic arteries currently used for myocardial revascularization.

The aim of this prospective randomized clinical trial was to compare the vascular reactivity of the internal thoracic arteries used for CABG depending on the type of pumps used (roller or centrifugal pump) and the impact of the SIRS generated during CPB on parietal inflammatory response of ITAs.

## Materials and methods

### 1. Study design (Fig 1)

The IPITA (Impact of Pumps on Internal Thoracic Arteries) study consisted of two parallel prospective, monocenter, randomized, active-treatment-controlled clinical trials. From November 3, 2015 to December 8, 2017, 80 patients were randomly assigned in a 1:1 ratio to two groups (RP group = Roller pump group; CFP group = Centrifugal pump group) one day before surgery in order to facilitate the perfusionist workup that consisted to install the adequate pump according to the randomization with use of a computer-generated plan. The randomization procedure was carried out by the Department of Methodology and Biostatistics at University Hospital of Angers. All patients provided their written informed consent preoperatively (S1 Appendix). The study and the consent form were approved by the Angers institutional review board (authorization: CNIL: 2015–002 (Commision Nationale de l’Informatique et des Libertés, December 7, 2015) (S2 Appendix). For each patient, the study began at the time of randomization and stopped at discharge of the hospital. No follow-up and no deviations from this study were planned. The authors confirm that all ongoing and related trails for this intervention were registered at ClinicalTrials.gov (Registration number: NCT04168853) after enrolment of participants started.

**Fig 1:**
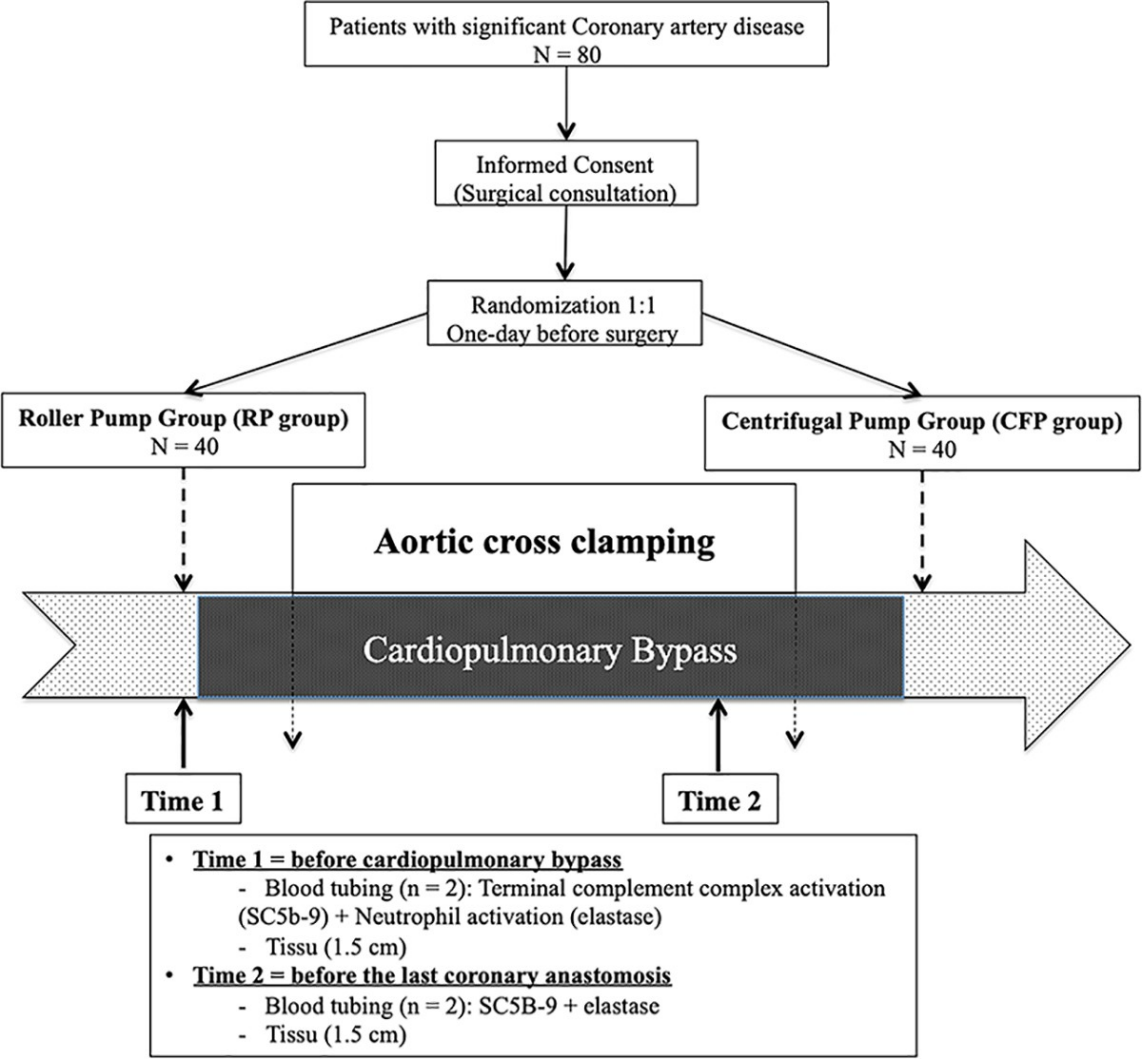
Flow-chart study.

The primary endpoint was the comparative measurement of internal thoracic artery (ITA) vascular reactivity, defined by endothelium-dependent relaxation rate. The secondary endpoint was the parietal inflammatory response of ITAs defined by superoxide detection, leukocytes and lymphocytes rate and gene expression of inflammatory proteins.

### 2.Population

Inclusion criteria were consecutive male patients and elective coronary artery bypass grafting (CABG) using at least one of the two ITAs.

Exclusion criteria were female patients because their complement activation has been shown to be greater than that in men during surgery under CPB [14]; age < 18 years; CABG requiring additional valve repair or replacement; emergency surgery and insufficient length of the ITA. Data were extracted from medical charts. Baseline characteristics, intraoperative and postoperative data were collected using Epidata 3.1 Software.

### 3. Surgical technique and ITAs segment sampling

- Surgical procedure: CABG was performed under normothermic (36–37°C) cardiopulmonary bypass (CPB). All components of the circuits were coated with phosphorylcholine inert surface (PHISIO, Sorin^®^). The pump manufacturers were Maquet^®^ and Sorin^®^ for the roller pumps and Sorin^®^ for the centrifugal pumps respectively. All patients underwent an hyperkalemic anterograde cold blood cardioplegia diluted with a 4:1 dilution Plegisol^®^ solution (Pfizer^®^). Heparin was administered after full sternotomy to obtain an activated clotting time (ACT) longer than 250 seconds as a routine procedure previously described in our center [15, 16]. The distal part of ITA was clamped and gathered after take-down and heparin administration.
- ITAs segment samples: 1.5 cm of ITA distality was gathered before blood flow interruption into the graft before starting CPB **(Time 1)** and another segment (1.5 cm) before the last coronary anastomosis during aortic cross clamping **(Time 2)** (Fig 1). Each arterial segment was cut into three parts: a fresh part for arterial myography bathed and stored in a 50 ml organ bath containing a physiological salt solution (PSS). The other two parts were cooled in liquid nitrogen and stored at -80°C for immunohistochemistry and RT-PCR analysis.

### 4. Internal thoracic arteries analysis

#### Myography

For each patient, 2 fresh segments of ITA (Time 1 and Time 2) stored in PSS were analyzed. On day+1, these segments were mounted on a wire-myograph (DMT, Aarhens, DK) [17]. Two tungsten wires (25 μm diameter) were inserted into the lumen of the arteries and connected to a force transducer and a micrometer, respectively. The arteries were bathed in the PSS solution. Wall tension, equivalent to intra-arterial pressure (90 mmHg), was applied and the blood vessels were allowed to stabilize for thirty minutes. Arterial contractility was assessed with phenylephrine (PE, 10 μmol/L). Acetylcholine-induced (Ach 10 μmol/L) relaxation was then obtained after phenylephrine-induced preconstruction (50% of maximal contraction) in the presence or in the absence of the NO synthesis blocker L-NMMA (3.10^−4^ mol/L) and in the presence or in the absence of the COX synthesis blocker Indomethacin (10^−5^ mol/L).

#### Superoxide detection and confocal microscopy

Dihydroethidium staining (DHE, Sigma-Aldrich) was used to evaluate the in-situ levels of superoxide anions (O_2_^-^). DHE is permeable to cells and is oxidized by superoxide (O_2_^-^) to fluorescent products that are trapped by intercalation into the DNA. Sections (10 μm thickness) were incubated with DHE (1 μmol/L) in phosphate-buffered solution (PBS) and DAPI (4’,6’-diamidino-2-phénulindole-Molecular probes, Invitrogen) for nuclear cells Fluorescent images of ethidium bromide were obtained using a confocal microscope (Nikon Eclipse TE2000S).

#### Immunochemistry

Sections (10 μm thickness) of arteries were rehydrated by 500 μl of PBS during 10 minutes and fixated with 200 μl of paraformaldehyde (PFA) (pH = 7.4, room temperature) and then were rinsed by PBS. Permeabilization with 200 μl of PBS-BSA (Bovine Serum Albumin–Sigma) 10%—Tween 0.1% during 40 minutes and then saturation with PBS-BSA 10% during 40 minutes were performed. Sections were incubated overnight with 100 μl of anti-CD45 antibody at 1/500th dilution for leukocyte staining or anti-CD80 antibody at 1/200th dilution for lymphocyte staining. Antibodies were labelled with a red fluorochrome (Phycoerythrine). Karyoplasm was stained blue by DAPI solution. On day+1, sections were rinsed with PBS and analyzed with the confocal microscope. Fluorescent images were quantified with the ImageJ (NIH) software.

#### Quantitative real time transcription-polymerase chain reaction (RT-PCR) analysis

Sections of ITAs were dried and stored at -80°C in RNA later Stabilization Reagent (Qiagen). RNA extraction was performed using the RNeasy^®^ micro kit (Qiagen). 500 ng of RNA extracted from each artery were used to synthesize cDNA using the QuantiTect^®^ Reverse Transcription kit (Qiagen). Quantitative real-time PCR was performed with Sybr^®^ Green PCR Master Mix (Applied Biosystems) using a Light cycler 480 Real-Time PCR System (Roche). (S1 Table)

### 5. Blood sampling and biochemical analysis

Serial arterial blood samples for SC5b-9, as a marker of terminal complement complex activation, and elastase and were collected at Time 1 and Time 2. Specimens were centrifuged (10 minutes, 3,000 rpm, 4°C) immediately to obtain plasma which was stored at– 80°C before analysis.

Enzyme-linked immunosorbent assay techniques were used from 10 μl of plasma to measure terminal complement (SC5b-9; Quidel, San Diego, CA, USA) and 50 μl of plasma for neutrophil elastase (Neutrophil ELA2, Assay pro, St Charles, USA). The limit of sensitivity of each assay undertaken was as follows: SC5b-9 = 16 ng/mL and elastase = 20 ng/mL.

### 6. Statistical analysis

All statistical analysis was done using SPSS software version 15.0 (SPSS, Chicago, IL, USA). Results were expressed as mean ± standard error of the mean (SEM) or mean and range. Significance of the difference between groups was determined by analysis of variance (two-way ANOVA for consecutive measurements followed by the Bonferroni *t-test*) to compare pressure-diameter curves in the different groups. We assumed a Gaussian distribution. Since the differences between paired values were consistent, a parametric paired *t-test* was used. P-values less than 0.05 were considered to be significant.

Sample size: There was no available data to predict the difference in vascular reactivity of ITAs according to the pumps. According to our previous reports comparing SIRS using CFP *vs* RP [4], vascular reactivity of cerebral arteries after CPB [13] and protection of vascular function from inflammation by pulsatility in resistance arteries of rat [9], we estimated that 20 patients per groups could be an adequate sample size to assess whether expected differences of SIRS would affect ITA reactivity. The myocardial revascularization surgical approach being left to surgeon discretion (in-situ *vs* composite Y anastomosis of both ITAs) and considering that both techniques in our routine practice would be roughly equally performed, we decided to include twice the amount of patients per group to compensate for potential withdrawal of patients due to different arterial grafts handling during aortic cross clamp time.

## Results

### 1. Baseline patient, surgical and postoperative course characteristics

Baseline patient and surgical characteristics are presented in Tables 1 and 2. Y-composite grafts were used in 16 patients of RP group and 19 patients in CFP group. Y-configuration grafting was performed according to the practice of surgeon’s usual practice and determined preoperatively. In this case, the segment of RITA was sampled just before the Y-anastomosis suture and before starting the CPB (Time 1).

**Table 1:** Characteristics of the study population.

Variables	RP group	CFP group
	n = 40	n = 40
Age (years)	66 [46–80]	67 [45–82]
Heigh (cm)	171 [158–187]	169 [152–187]
Weight (kg)	79 [56–113]	80 [56–117]
BMI (kg/m^2^)	27 [20–35]	28 [20–37]
Diabetes mellitus, n (%)	12 (30)	9 (23)
Hypertension, n (%)	32 (80)	25 (64)
Dyslipidemia, n (%)	29 (72)	29 (72)
Current smoking, n (%)	6 (15)	4 (10)
Previous AMI	9 (22)	9 (22)
1 vessel, n (%)	4 (10)	1 (2.5)
2 vessels, n (%)	12 (30)	17 (44)
3 vessels, n (%)	24 (60)	22 (55)
Logistic Euroscore 1 (%)	2.15 [1–3.9]	2.6 [0.5–8.4]
LVEF (%)	58 [35–74]	56 [30–77]
Platelet inhibitors, n (%)	34 (85)	36 (90)
β-blockers, n (%)	35 (87)	33 (84)
ACE-inhibitors, n (%)	29 (72)	27 (69)

BMI, body mass index; AMI, acute myocardial infarction; LVEF, left ventricular ejection; ACE, angiotensin-converting enzyme. Data are presented as mean [min-max] or n (%)

**Table 2:** Surgical data.

Variables	RP group	CFP group
	n = 40	n = 40
Anastomosis per patients n (%)	2.5 [1–5]	2.5 [1–5]
Conduits		
LITA n (%)	40 (100)	40 (100)
BITA n (%)	27 (67)	26 (65)
SVG n (%)	15 (37)	17 (42)
Y-composite graft n (%)	16 (40)	19 (49)
Sequential grafts		
With LITA n (%)	9 (22)	9 (22)
With RITA n (%)	5 (12)	6 (15)
Cardiopulmonary bypass duration (min)	87 [46–158]	94 [41–184]
Aortic cross-clamping time (min)	61 [23–127]	68 [23–144]
Heparin dose (mg)	157 [50–250]	172 [12–360]
Minimum ACT (sec)	217 [122–250]	222 [122–250]
Maximum ACT (sec)	303 [254–374]	303 [254–442]
Exposure time of ITA to the pump flow (min)	43.3 [11–135]	45.7 [11–135]

LITA, Left internal thoracic artery; BITA, Bilateral internal thoracic artery; SVG, Saphenous vein graft; ACT, Activated Clotting Time. Data are presented as mean [min-max] or n (%).

The duration between Time 1 and Time 2 was 43.3 minutes [11–135] in RP group and 45.7 minutes [11–135] in CFP group.

### 2. Endothelium-dependent relaxation of internal thoracic arteries

**Fig 2:**
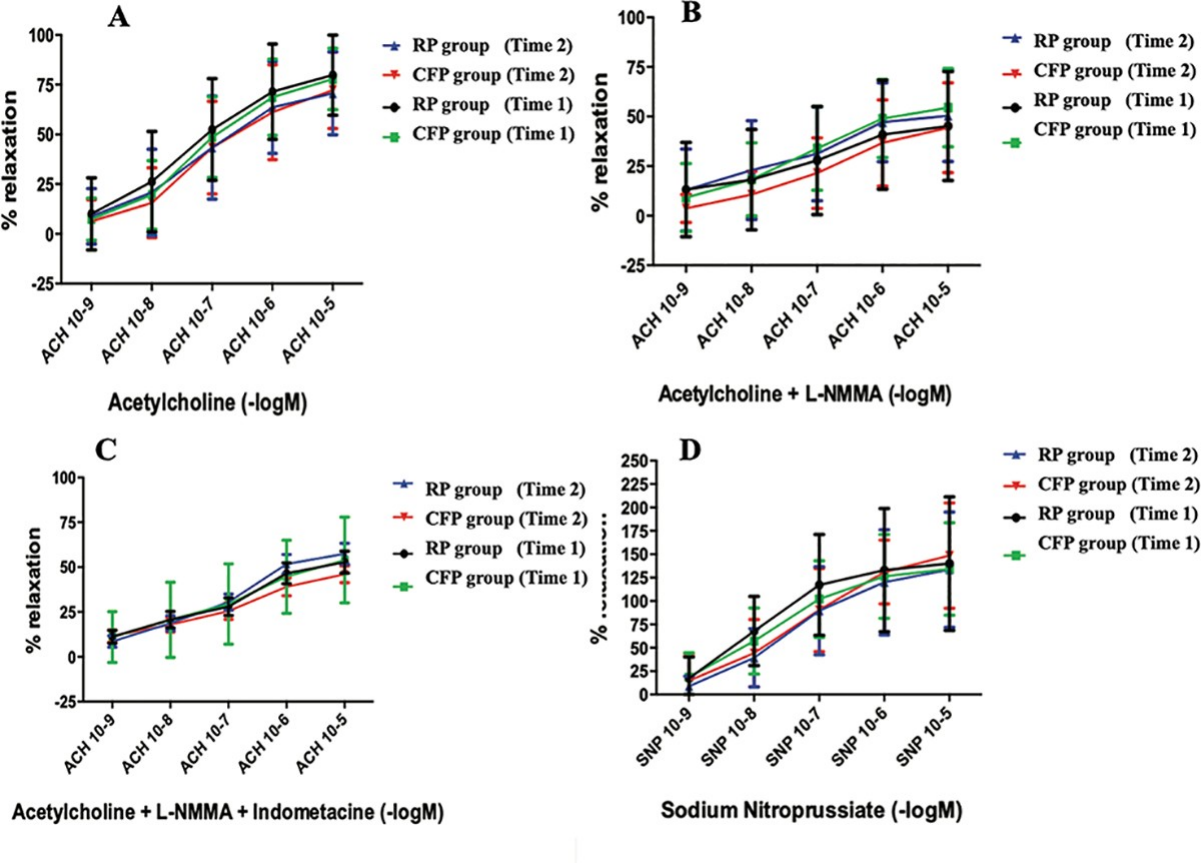
Myography results with Y-graft configuration. Dilatation induced by increasing dose of Acetylcholine in phenylephrine pre-constricted artery (A). Reduction of acetylcholine-dependent dilatation with NO synthesis blockade L-NMMA (N-monomethyl-L-Arginine) (B) then Indomethacin (C) was observed. Dilatation was induced by increasing the dose of sodium nitroprusside (D). Ach, acetylcholine; SNP, sodium nitroprusside; Data are presented as mean ± SEM.

**Fig 3:**
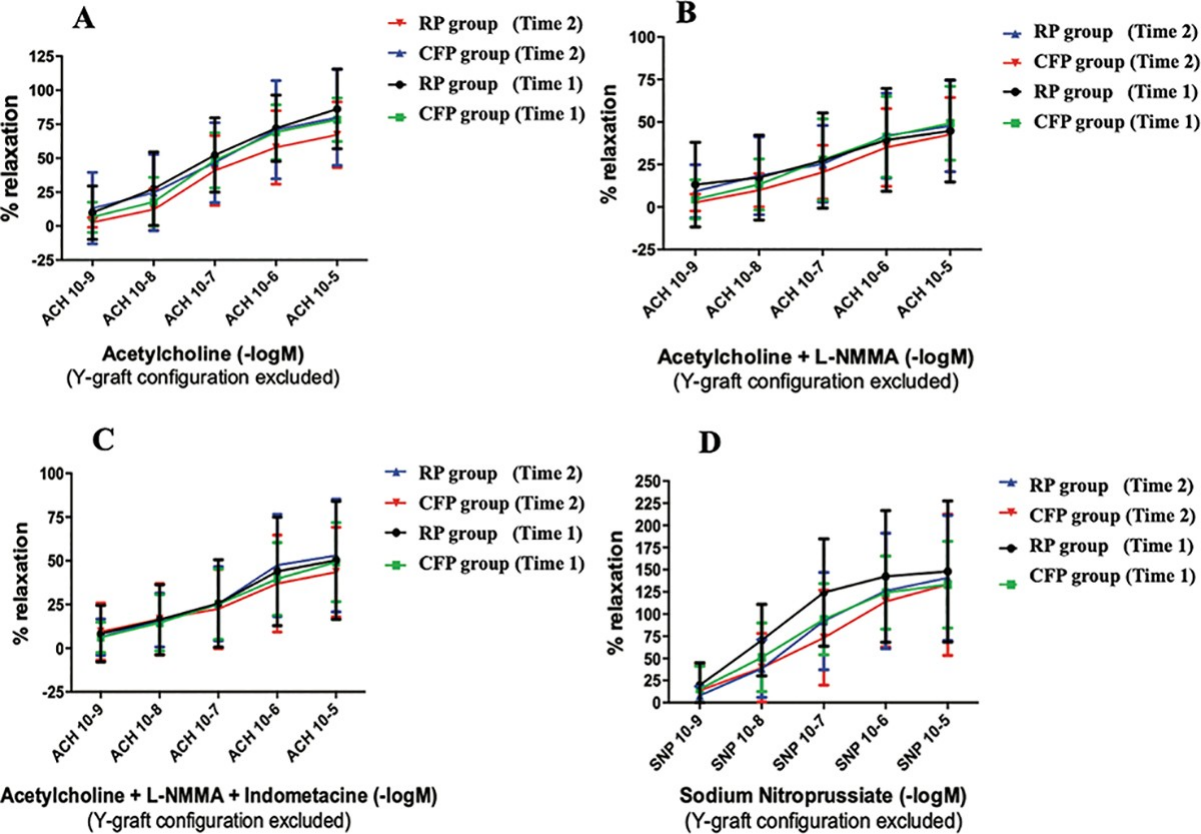
Myography results without Y-graft configuration.

Acetylcholine-dependent relaxation was observed in both groups regardless of the time (before CPB = time 1 or before coronary anastomosis = time 2). In both groups, the dilatation induced by acetylcholine stepwise in ITAs of RP group was equivalent to ITAs in CFP group (Fig 2). NO synthesis blockade with L-NMMA reduced acetylcholine-dependent dilatation in all groups. Sodium nitroprusside induced dilatation in the ITAs, which was not affected by flow characteristics. The same observation was found when Y-graft configuration was excluded (Fig 3).

### 3. Molecular biology (qT-PCR)

**Fig 4:**
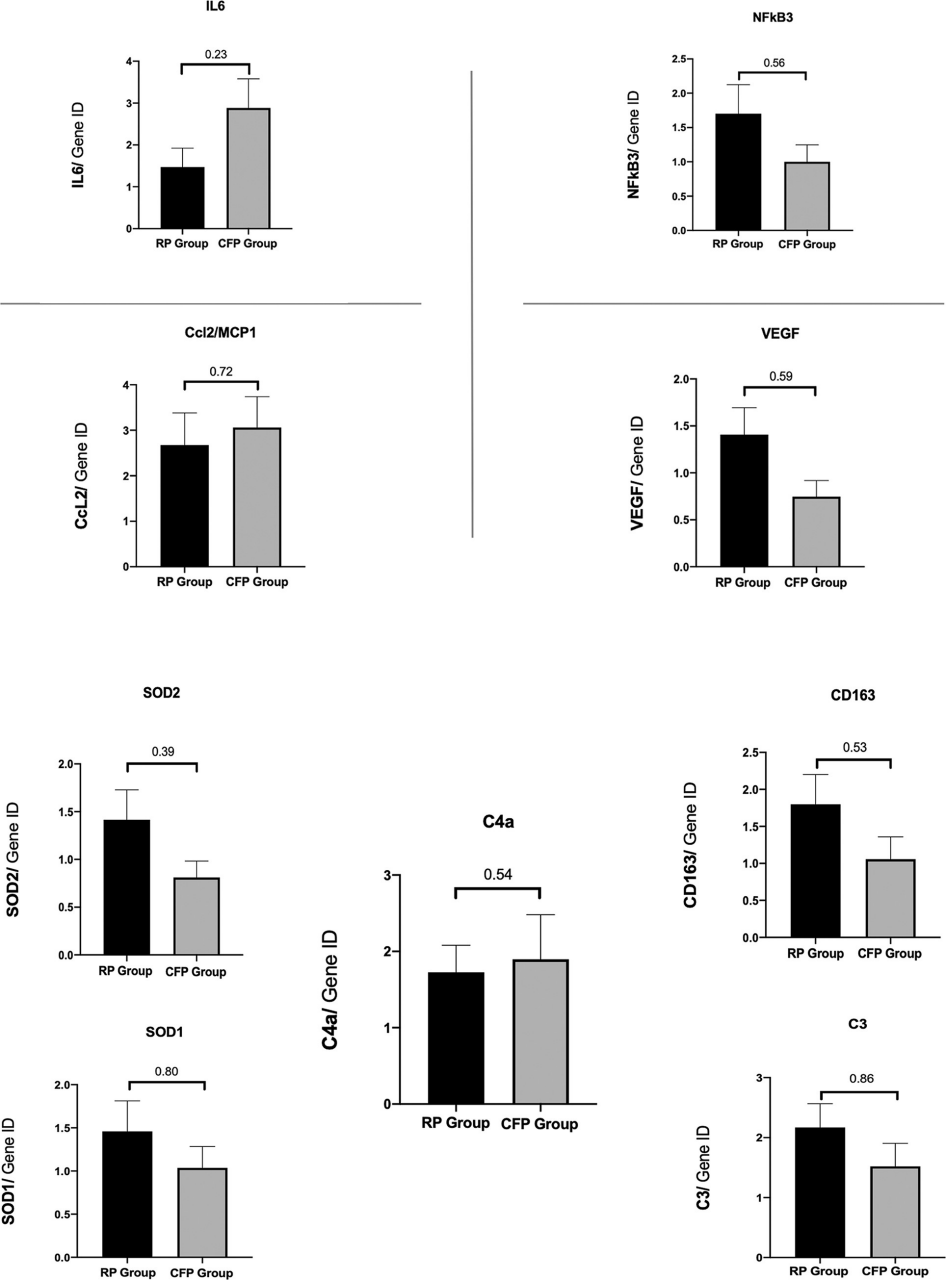
Protein expression levels from RP group to CFP group at time 1 or time 2 in artery sections. Expression of genes coding: cytokines, IL-6 (Interleukin-6), MCP-1 (Monocyte Chemotactic protein 1), CCL2; growth and transcription factors, VGEF (Vascular Growth Endothelial Factor), NF_k_B3 (pro-inflammatory transcription factor) **(Fig 4A)**. CD163, macrophages; oxidative stress, SOD1 and SOD2 (superoxide dismutase 1 and 2), complement complex, C3 and C4a **(Fig 4B)**. Data are presented as mean ± SEM (*Time 2-Time 1* for RP and CFP groups).

RNA extraction was performed in patients without Y-configuration ITAs or samples without vascular reactivity observed during myography (N = 80 analyzed segments corresponding to 21 and 19 patients respectively in the RP group and the CFP group). There was no difference between groups concerning macrophages and lymphocytes markers, oxidative stress, mitochondrial activity, growth and transcription factors, pro-inflammatory factors (Fig 4A and 4B). Gene expression for C3 complement decreased from Time 1 (0.21±0.02) to Time 2 (0.14±0.02) (p = 0.01) for RP group. Gene coding C4a complement decreased from Time 1 to Time 2 for both group (p<0.05).

### 4. Immunochemistry

**Fig 5:**
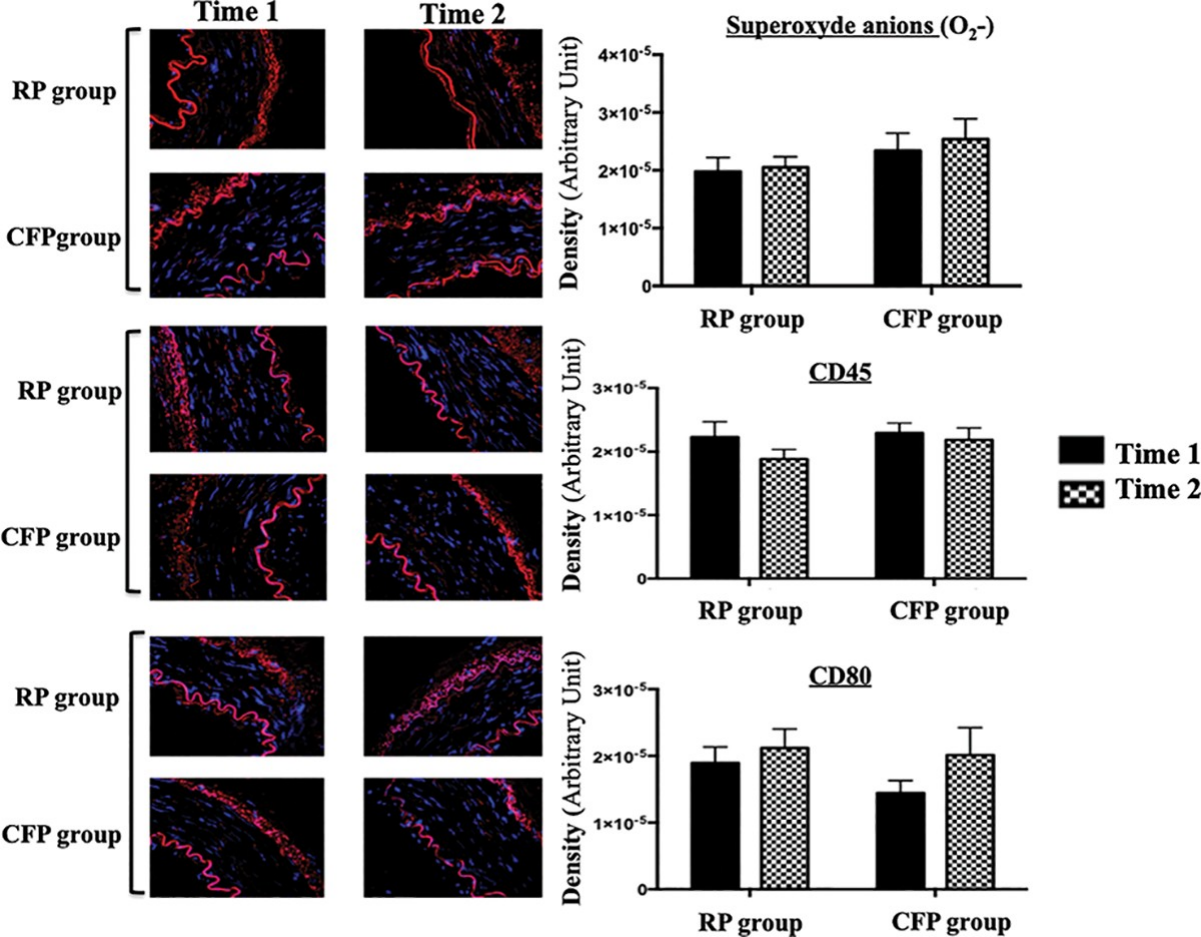
In situ detection of superoxide anion, CD 45 and CD80 in RP group and CFP group from Time 1 to Time 2 are shown in the bar graph.

No significant difference in superoxide anions level was detected between RP group and CFP group and between Time 1 and 2. The same findings were observed for anti-CD45 and anti-CD80 antibody level (Fig 5).

### 5. Terminal complement complex activation

**Fig 6:**
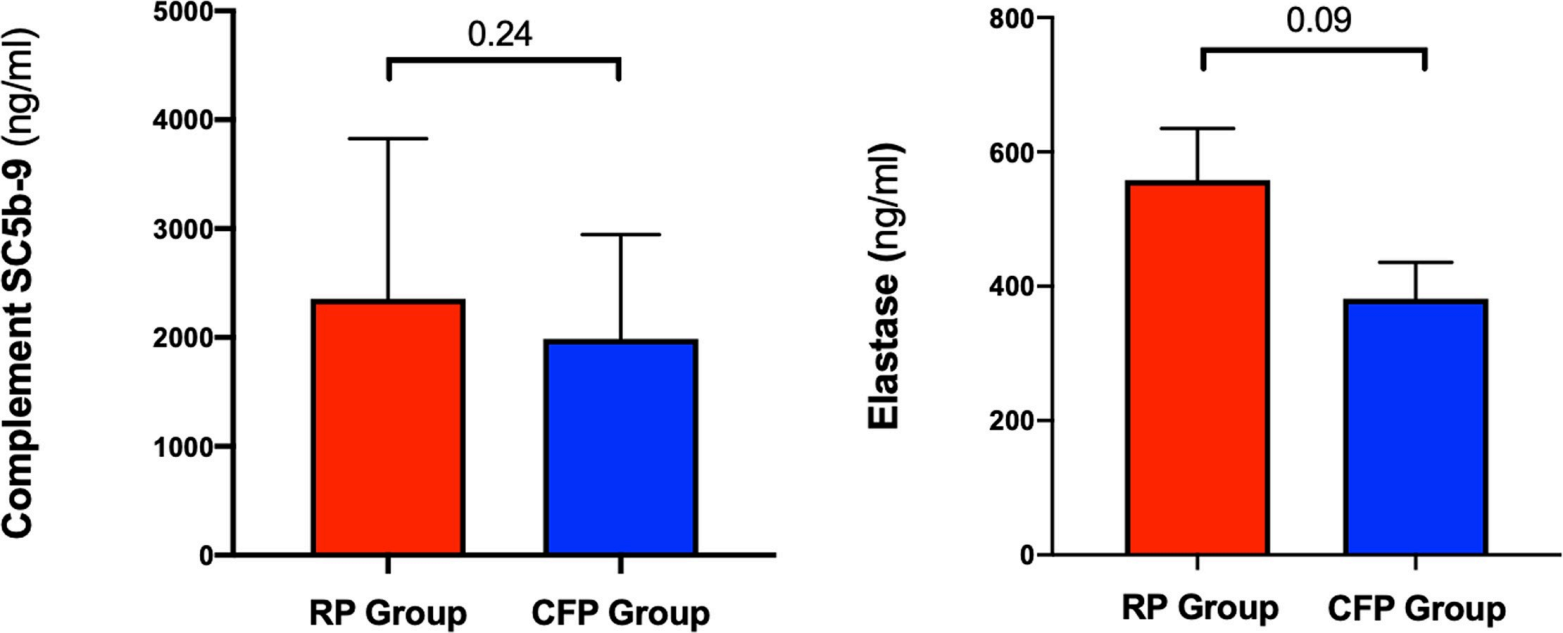
Complement activation SC5b-9 (Fig 6A) and elastase (Fig 6B) release in the blood are shown in the bar graph for each group (*Time 2-Time 1)*. Data are presented as mean ± SEM.

Significant increases in C5b-9 levels were observed in both groups at Time 2 (= before coronary anastomosis) compared to Time 1 (= before starting CPB). The mean value of C5b-9 was 179±10.9 ng/ml at Time 1 and 2533±235.2 at Time 2 in the RP group (p<0.0001) and 183.5±14.16 ng/ml and 2222±146.5 in the CFP group. No difference was observed between the RP group and the CFP group (Fig 6A).

### 6. Neutrophil activation

In both groups, elastase levels at Time 2 were increased compared to Time 1. The mean value of elastase at Time 1 was 196.5±13.24 ng/ml and 754.5±74.81 at Time 2 in the RP group (p<0.0001) and 182.2±19.04 ng/ml and 565.2±59.45 in the CFP group (p<0.0001). No difference was observed between the RP and the CFP groups (Fig 6B).

## Discussion

Systemic inflammatory response syndrome (SIRS) after CPB is considered as an important cause of postoperative events in coronary surgery [18]. The indications for myocardial revascularization of left main coronary artery for stable coronary artery disease were in accordance with the 2018 ESC/EACTS guidelines in our center [19]. The exponential growth in percutaneous coronary intervention procedures due to the results of randomized studies comparing long-term clinical outcomes of CABG or Stent-Percutaneous Coronary Intervention (PCI) [20] has led to operate on patients with more co-morbidities and more advanced cardiac disorders, who are even more sensitive to inflammatory response after CPB [21]. There are many causes of inflammatory response and they have been extensively described: surgical trauma, contact between blood and artificial surfaces of CPB equipment, activation of coagulation, cellular and humoral cascade attenuation, hemodilution and uncontrolled suction of pericardial shed blood [22]. The early inflammatory response observed immediately after CPB start, as reflected by markers released during the procedure, is known to affect the complement system and neutrophils [23]. However, no difference was observed between RP and CP unlike data in the literature [16, 24], probably because the blood samples were collected at the end of aortic cross clamp time, before CPB arrest. Moreover, compared to historical comparisons [4, 5], we used a tubing set improved by phosphorylcholine coated surface as well as a cell saver was systematically used to discard highly activated blood from pericardial cavity for better biocompatibility. Hypothermic blood cardioplegia, as used in our study, was associated with a decrease proinflammatory cytokines (IL-6) in the early postoperative period and anti-inflammatory cytokine (IL-10) compared to normothermic blood cardioplegia [25].

Others experimental studies have shown the impact of CPB on vascular reactivity of small diameter arteries. Early and transient mesenteric or cerebral endothelial dysfunction occurred quickly after starting CPB [12, 26]. CPB and cardioplegic arrest affected the vascular bed, reduced vascular resistance in skeletal muscle and increased the risk of vasospasm in the pulmonary, cardiac, mesenteric and cerebral microvascularization [12].

The debate about the role of pulsatile or non-pulsatile CPB and its impact on the microcirculation has been ongoing for several decades. Roller pump-generated pulsatile flow has been described as « ripple flow » corresponding to the variation in the flow rate of the pump which is different in the centrifugal pump [27]. The microcirculation corresponding to blood vessels with an internal diameter of less than 200 μm is preserved with a pulsatile perfusion compared to non-pulsatile flow because of leukocyte adherence attenuation and the maintenance of the number of perfused vessels [28]. In resistance arteries, such as the mesenteric arteries in rats, pulsatile pressure induced significant dilatation and stepwise increases in pressure induced contraction but less so under non-pulsatile condition [9]. An inflammatory response in the artery wall with a significant ROS level (appearing from 30 minutes), MCP-1 and TNF-α production were significant in arteries subjected to non-pulsatile flow [9]. Regarding these results, it appeared useful to verify if human small arteries such as ITAs, the principal graft conduit used in coronary surgery, alter their vascular reactivity, an effect that we did not observe in the present study. Use of pulsatile CPB attenuates leakage of endothelial markers (vascular endothelial growth factor (VGEF), monocyte chemo-attractant protein (MCP-1) and cytokines (IL-10 and IL-2) [29].

We investigated a wide range of genes coding for the classical and alternative pathways of the complement terminal complex (components C3 and C4a), macrophages (CD163), lymphocytes (PTPRC), mitochondrial activity and cytokines. We observed a decrease in C3 and C4a components at time 2. C3 and C4a components were anaphylatoxin molecules and mediated two major effects, namely enhancement of vascular permeability and induction of smooth muscle contraction [30]. A decrease in gene expression of complement, observed in the present study, could be explained by intravascular complement system activation. An increase in oxidative stress was not found in ITAs contrary to the results of experimental studies [9]. Exposure duration to the flow was relatively short around 45 minutes. A direct correlation between cytokine production and CPB duration, consequently associated with adverse outcomes such as longer stay in the intensive care unit and longer duration mechanical ventilation have been clearly established [28], with altered vascular reactivity during non-pulsatile perfusion starting from 180 minutes of CPB [9].

Use of circulating microRNAse as endothelial miR-16 to monitor the inflammatory impact during extracorporeal circulation was probably an excellent biomarker. Sorrentino et al. demonstrated an increase of miR-16, miR-155, miR-34 and miR-17 expression levels in carotid artery endothelium 14 days from injury. Circulating miR-16 was upregulated after vascular injury in the presence of hindlimb ischemia [31] and exerted a negative effect on vascular remodeling. However, the detection of miRNA required a long period of stimulation or inflammation duration corresponding to vascular injury from several days to several weeks [31, 32]. In our study, the duration of CPB was respectively 87 minutes [46–158] and 94 [41–184] in both groups and was probably too short to detect circulating miR-16.

Perioperative hemodynamic monitoring including measurement of mean arterial pressure, cardiac index and mean pulmonary pressure classically is performed during cardiac surgery. However, there was no quantification of « pulsatile rate » in our study; this parameter (energy equivalent pressure (EEP) and surplus hemodynamic energy (SHE) according to Shepard’s models [33]) could vary during CPB.

The main limitation of this study was the evolving surgical technique concerning the arterial graft including Y-graft configuration according to the debate that exists with cardiologists about the improved results expected by the use of multiple arterial grafts. In addition, manipulation of Y composite grafts may expose to disturbed vascular reactivity injury due to local ischemia-reperfusion injury.

## Conclusion

Within the time frame necessary to perform regular CABG in the IPITA study, roller versus centrifugal pumps did not alter endothelium-dependent relaxation of the internal thoracic arteries because no parietal inflammatory response of ITAs appeared despite the initiation of the systemic inflammatory response during CPB.

## Supporting information

S1 Checklist CONSORT 2010 checklist of information to include when reporting a randomised trial*. (DOC)

S1 Table Primer pairs were designed using 3 reference genes and those presenting a single peak of dissociation and an efficacy were retained. (DOCX)

S1 Fig CONSORT 2010 flow diagram. (DOC)

S1 Appendix The original study protocol in French; translation of the study protocol in English. (DOC)

S2 Appendix The ethics committee approved by the Angers institutional review board (authorization: CNIL: 2015–002). (PDF)

S3 Appendix (DOC)

S4 Appendix (DOC)

## References

[1] Database French Society of Thoracic and CardioVascular Society 2014

[2] Haines N, Wang S, Undar A, Alkan T, Akcevin A. Clinical outcomes of Pulsatile and Non-pulsatile mode of perfusion. J extra Corpor Technol. Mars 2009;41(1):p26–9 PMC4680229 19361037

[3] Saczkowski R, Maklin M, Mesana T, Boodhwani M, Ruel M. Centrifugal pump and roller pump in adult cardiac surgery: a meta-analysis of randomized controlled trials. Artif Organs. 2012 8 1;36(8):668–76. 10.1111/j.1525-1594.2012.01497.x 22804106

[4] Ashraf S, Butler J, Tian Y, Cowan D, Lintin S, Saunders NR, et al Inflammatory mediators in adults undergoing cardiopulmonary bypass: comparison of centrifugal and roller pumps. Ann Thorac Surg. 1998 2;65(2):480–4. 10.1016/s0003-4975(97)01349-0 9485250

[5] Baufreton C, Intrator L, Jansen PG, Velthuis te H, Le Besnerais P, Vonk A, et al Inflammatory response to cardiopulmonary bypass using roller or centrifugal pumps. Ann Thorac Surg. 1999 4;67(4):972–7. 10.1016/s0003-4975(98)01345-9 10320237

[6] James SA, Peters J, Maresca L, Kalush SL, Trigueros EA. The roller pump does produce pulsatile flow. J Extra Corpor Technol. 1987 10 27;19:376–83.

[7] Wright G. The hydraulic power outputs of pulsatile and nonpulsatile cardiopulmonary bypass pumps. Perfusion. SAGE Publications; 1988;3(4):251–62.

[8] Watarida S, Mori A, Onoe M, Tabata R, Shiraishi S, Sugita T, et al A clinical study on the effects of pulsatile cardiopulmonary bypass on the blood endotoxin levels. J Thorac Cardiovasc Surg. 1994 10;108(4):620–5. 7934094

[9] Pinaud F, Loufrani L, Toutain B, Lambert D, Vandekerckhove L, Henrion D, et al In vitro protection of vascular function from oxidative stress and inflammation by pulsatility in resistance arteries. J Thorac Cardiovasc Surg. 2011 11;142(5):1254–62. 10.1016/j.jtcvs.2011.07.007 21843894

[10] Crow S, John R, Boyle A, Shumway S, Liao K, Colvin-Adams M, et al Gastrointestinal bleeding rates in recipients of nonpulsatile and pulsatile left ventricular assist devices. J Thorac Cardiovasc Surg. 2009;137(1):208–15. 10.1016/j.jtcvs.2008.07.032 19154927

[11] Bartoli CR, Restle DJ, Zhang DM, Acker MA, Atluri P. Pathologic von Willebrand factor degradation with a left ventricular assist device occurs via two distinct mechanisms: Mechanical demolition and enzymatic cleavage. J Thorac Cardiovasc Surg. 2015 1;149(1):281–9. 10.1016/j.jtcvs.2014.09.031 25439775

[12] Ruel M, Khan TA, Voisine P, Bianchi C, Sellke FW. Vasomotor dysfunction after cardiac surgery. Eur J Cardiothorac Surg. 2004 11 1;26(5):1002–14. 10.1016/j.ejcts.2004.07.040 15519196

[13] Baufreton C, Pinaud F, Corbeau J-J, Chevailler A, Jolivot D, Minassian Ter A, et al Increased cerebral blood flow velocities assessed by transcranial Doppler examination is associated with complement activation after cardiopulmonary bypass. Perfusion. 2011 3;26(2):91–8. 10.1177/0267659110392439 21173036

[14] Baufreton C, Jansen PG, Le Besnerais P, te Velthuis H, Thijs CM, Wildevuur CR et al Heparin coating with aprotinin reduces blood activation during coronary artery operations. Ann Thorac Surg. 1997 1;63(1):50–6. 10.1016/s0003-4975(96)00964-2 8993240

[15] Baufreton C, De Brux JL, Binuani P, Corbeau JJ, Subayi JB, Daniel JC et al A combined approach for improving bypass in coronary surgery: a pilot study. Perfusion 2002 11;17(6):407–13. 10.1191/0267659102pf615oa 12470029

[16] Shapira OM, Korach A, Pinaud F, Dabah A, Bao A, Corbeau JJ et al Safety and efficacy of biocompatible perfusion strategy in a contemporary series of patients undergoing coronary artery bypass grafting: a two-center study. J Cardiothorac Surg. 2014 12 18;9: 196 10.1186/s13019-014-0196-3 25519179 PMC4274677

[17] Henrion D., Laher I and Bevan JA. Intraluminal flow increases vascular tone and 45 Ca^2+^ in flux in the rabbit fascial vein. Circulation Research. 1992, vol.71, no 2, pp. 339–345. 10.1161/01.res.71.2.339 1628391

[18] John W, Hammon. Extracorporeal circulation: the response of humoral and cellular elements of blood extracorporeal circulation In: Cohn LH, editor. Cardiac surgery in the adult, vol.3 New-York: McGraw-Hill 2008 p.370–89.

[19] Neumann FJ, Sousa-Uva M, Ahlsson A, Alfonso F, Banning AP, Benedetto U, et al 2018 ESC/EACTS Guidelines on myocardial revascularization. Eur Heart J. 2019 1 7;40(2):87–165. 10.1093/eurheartj/ehy394 30165437

[20] De Rosa S, Polimeni A, Sabatino J, Indolfi C.Long-term outcomes of coronary artery bypass grafting versus stent-PCI for unprotected left main disease: a meta-analysis. BMC Cardiovasc Disord. 2017 9 6;17(1):240 10.1186/s12872-017-0664-5 28877676 PMC5588710

[21] Casthelheim A, Hoel TN, Vedem V et al Biomarker profile in off-pump coronary artery bypass grafting surgery in low-risk patients. Ann Thorac Surg 2008;85:1994–2002. 10.1016/j.athoracsur.2008.03.012 18498809

[22] Onorati F, Esposito A, Comi MC et al Intra-aortic ballon pump-induced pulsatile flow reduces coagulative and fibrinolytic response to cardiopulmonary bypass. Artif Organs 2008; 32:433–41. 10.1111/j.1525-1594.2008.00563.x 18422802

[23] Boyle EM, Pohlman TH, Johnson MC, Verrier ED. Endothelial cell injury in cardiovascular response. Ann Thorac Surg 1997; 63:277–84. 10.1016/s0003-4975(96)01061-2 8993292

[24] Baufreton C, Intrator L, Jansen PGM et al Inflammatory response to cardiopulmonary bypass using roller or centrifugal pumps. Ann Thorac Surg. 1 Avr 1999;67(4):972–7 10.1016/s0003-4975(98)01345-9 10320237

[25] Sacli H, Kara I, Diler MS, Percin B, Turan AI, Kirali K. The relationship between the use of cold and isothermic blood cardioplegia solution for myocardial protection during cardiopulmonary bypass and the ischemia-reperfusion injury. Ann Thorac Cardiovasc Surg. 2019 7 12. 10.5761/atcs.oa.18-00293 PMC6923728 31308305

[26] Doguet F, Litzler PY, Tamion F et al Changes in mesenteric vascular reactivity and inflammatory response after cardiopulmonary bypass in a rat model. Ann Thorac Surg. 2004 6;77(6):2130–7 10.1016/j.athoracsur.2003.10.034 15172281

[27] Sunagawa G, Koprivanac M, Karimov JH, Moazami N, Fukamachi K. Is a pulse absolutely necessary during cardiopulmonary bypass ? Expert Rev Med Devices. 2017 1;14(1):27–35. 10.1080/17434440.2017.1265445 27892719

[28] O'Neil MP, Fleming JC, Badhwar A, Guo L. Pulsatile versus nonpulsatile flow during cardiopulmonary bypass: microcirculatory and systemic effects. Ann Thorac Surg. 2012 12;94(6):2046–53. 10.1016/j.athoracsur.2012.05.065 22835552

[29] Onorati F, Rubino AS, Nucera S et al Off-pump coronary artery bypass surgery versus standard linear or pulsatile cardiopulmonary bypass: endothelial activation and inflammatory response. Eur J Cardiothorac Surg. 2010 4; 37(4):897–904. 20018523 10.1016/j.ejcts.2009.11.010

[30] Hugli TE. Structure and function of the anaphylatoxins. Springer Semin Immunopathol. 1984;7(2–3):193–219. Review. 10.1007/BF01893020 6387982

[31] Sorrentino S, Iaconetti C, De Rosa S, Polimeni A, Sabatino J, Gareri C, et al Hindlimb Ischemia Impairs Endothelial Recovery and Increases Neointimal Proliferation in the Carotid Artery. Sci Rep. 2018 1 15;8(1):761 10.1038/s41598-017-19136-6 29335599 PMC5768880

[32] Farina FM, Hall IF, Serio S, Zani SM, Climent M, Salvarani Net al. miR-128-3p Is a Novel Regulator of Vascular Smooth Muscle Cell Phenotypic Switch and Vascular Diseases. Circ Res. 2020 3 27. 10.1161/CIRCRESAHA.120.316489 32216529

[33] Shepard RB, Simpson DC, Sharp JF. Energy equivalent pressure. Arch Surg 1966;93:730–40. 10.1001/archsurg.1966.01330050034005 5921294

