# Supplementary material for: Cardiopulmonary bypass and internal thoracic artery: Can roller or centrifugal pumps change vascular reactivity of the graft? The IPITA study: A randomized controlled clinical trial
Source: PLoS One. 2020 Jul 9;15(7):e0235604. doi: 10.1371/journal.pone.0235604 (PMC7347139; doi:10.1371/journal.pone.0235604)
Supplement: S1 Table — (DOCX) [file pone.0235604.s002.docx]

**SUPPLEMENT**

| **Gene** | **Protein** | **Gene ID** | **Primers sequence** | |
| --- | --- | --- | --- | --- |
|  |  |  | **Forward** | **Reverse** |
|  |  |  |  |  |
| **ACTB** | Beta actin | NM_001101.3 | gccagggcttacctgtacact | catttttaaggtgtgcacttttattc |
| **GAPDH** | GAPDH | NM_002046.3 | agccacatcgctcagacac | gcccaatacgaccaaaatcc |
| **CD163** | CD163 | NM_004244.5 | gaagatgctggcgtgacat | gctgcctccacctctaagtc |
| **CD3g** | CD3g | NM_000073.2 | tccttgctgttggggtctac | cagagtctgcttgtctgaagctc |
| **SOD1** | Superoxide dismutase 1 | NM_000454.4 | ccaccgtgttttctggata | tccatgttcatgagtttggagat |
| **SOD2** | Superoxide dismutase 2 | NM_001322816.1 | gcactagcagcatgttgagc | ccgtagtcgtagggcaggt |
| **C4a** | C4a complement | NM_007293.2 | ttctgccacggtgtcttctc | agaatccggccgctcaatag |
| **C3** | C3 complement | NM_000064.3 | tgtctagctttcaaagttcaccaa | gaaccgggtacagctttcct |
| **PTPRC** | PTPRC | NM_002838.4 | accaggaatggatgtcgcta | tggggcctgtaaaagtgcc |
| **PPARGC1A** | PPARG coactivator 1 alpha | NM_001354825.1 | tgagagggccaagcaaag | ataaatcacacggcgctctt |
| **TFAM** | Transcription factor A, mitochondrial | NM_001270782.1 | gaacaactacccatatttaaagctca | gaatcaggaagttccctcca |
| **IL2** | IL2 | NM_000586.3 | aagttttacatgcccaagaagg | aagtgaaagttttgctttgagcta |
