# Supplementary material for: Cardiopulmonary bypass and internal thoracic artery: Can roller or centrifugal pumps change vascular reactivity of the graft? The IPITA study: A randomized controlled clinical trial
Source: PLoS One. 2020 Jul 9;15(7):e0235604. doi: 10.1371/journal.pone.0235604 (PMC7347139; doi:10.1371/journal.pone.0235604)
Supplement: S1 Fig — (DOC) [file pone.0235604.s003.doc]

**
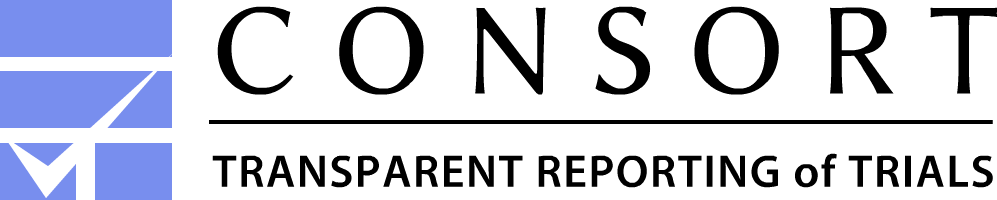
**

**CONSORT 2010 Flow Diagram**

**Allocation**

**Analysis**

**Follow-Up**

**Enrollment**

Assessed for eligibility (n=80 )

Excluded (n= 0 )

♦  Not meeting inclusion criteria (n= )

♦  Declined to participate (n= )

♦  Other reasons (n= )

Analysed (n=40 )
♦ Excluded from analysis (give reasons) (n= 0 )

Lost to follow-up (give reasons) (n=0 )

Discontinued intervention (give reasons) (n= 0 )

Allocated to intervention (n=40 )

♦ Received allocated intervention (n=40 )

♦ Did not receive allocated intervention (give reasons) (n= 0 )

Lost to follow-up (give reasons) (n=0 )

Discontinued intervention (give reasons) (n= 0)

Allocated to intervention (n=40 )

♦ Received allocated intervention (n= 40 )

♦ Did not receive allocated intervention (give reasons) (n= 0 )

Analysed (n=40 )
♦ Excluded from analysis (give reasons) (n= 0 )

Randomized (n= 80 )
