# Supplementary material for: Cardiopulmonary bypass and internal thoracic artery: Can roller or centrifugal pumps change vascular reactivity of the graft? The IPITA study: A randomized controlled clinical trial
Source: PLoS One. 2020 Jul 9;15(7):e0235604. doi: 10.1371/journal.pone.0235604 (PMC7347139; doi:10.1371/journal.pone.0235604)
Supplement: S1 Appendix — (DOC) [file pone.0235604.s004.doc]

**Impact of Pulsatility on Internal Thoracic Arteries**

**IPITA**

**Abstract**

| Administrator | CHU Angers (Angers University Hospital) |
| --- | --- |
| Principal investigator researcher (Coordinator): | Dr. Olivier Fouquet  Cardiac Surgery Department  CHU Angers, 49933 Angers cedex 09 |
| Protocol version | **Error: Reference source not found**09/09/2019 |
| Rationale/background | Cardiovascular conditions are the leading cause of mortality with our aging population increasing their significance. There are estimated to be almost 15,000 surgical coronary revascularisation procedures every year. The vast majority of patients will have an internal thoracic artery used as a graft to revascularize one of the principle coronary arteries. The use of extracorporeal circulation affects 90 % of the procedures and requires a pulsated or non-pulsated flow rate depending on the type of arterial pump. The absence of pulsatility leads to an increase in the inflammatory response and a deterioration of myogenic tone in microcirculation as has been shown in the experimental studies. We are yet to understand the impact of the absence or otherwise presence of pulsatility on arterial grafts and the effect that they could may have on their permeability. |
| General objective | To assess and compare the impact of 2 types of pumps used in routine ECC practice (roller and centrifugal pumps) on the endothelial function of the ITAs required for coronary bypass surgery. |
| Objectives | - Study the vascular reactivity of the internal thoracic artery according to what type of pump is used (pulsatile or non-pulsatile) - Study of the parietal inflammatory response in relation to pulsatility - Study of the general inflammatory response in relation to pulsatility |
| Assessment Criterion | Study of the vascular reactivity of the internal thoracic artery:   - Vasodilation (dependent on dilation flow rate): Flow-mediated dilation percentage - Myogenic tone: Contraction percentage in relation to pressure   Measurement of parietal and systemic oxidative stress |
| Methodology/Study plan | Interventional clinical study in routine care, prospective, randomised, monocentric, aimed at assessing two class IIb medical devices.  The study, carried out during ECC with a centrifugal (non-pulsatile flow) or roller pump (pulsatile flow), compares the impact on endothelial viability and function of the internal thoracic arterial grafts.  Two groups of patients: group 1 (centrifugal pump), group 2 (roller pump).  In each group, samples of artery segments are taken before coronary anastomosis:   - Before ECC: the artery is subjected to cardiac output - During the aortic clamping, before anastomosis: the artery is subjected to the flow of the arterial pump   Tests carried out on the arterial segments: vascular reactivity, mechanotransduction, histology, immunohistochemistry, biochemistry  In each group, the blood samplings will be carried out before the start of ECC and during the clamping, sampling carried out just before the arterial segment is removed:   - Sc5b-9 - Leukocyte Elastase |
| Patient Inclusion Criteria | Patients aged over 18 years old  Male  Elective coronary bypass using at least one internal thoracic artery |
| Patient Non-Inclusion Criteria | Emergency surgery  Female  Combined surgery  Participation in another protocol |
| Devices/Strategies/Procedures | In routine practice, the pump choice, centrifugal or roller, is decided at random.  For the purposes of the study, the type of pump used will depend on the randomisation, the surgical technique will not be changed. |
| Number of patients | 80 patients |
| Study duration | Inclusion period duration: 2 years  Study duration for a patient: approx. 1 month  Total study duration: 2 years and 1 month |
| Expected benefits | To assess the benefit of maintaining pulsed flow during ECC in terms of myogenic tone deterioration and endothelial cell damage in ITAs.  To provide additional arguments for a recommendation for the use of roller pumps during coronary bypass surgery. |

**List of abbreviations**

| MA | Marketing authorisation |
| --- | --- |
| CRA | Clinical research associate: |
| LITA | Left internal thoracic artery |
| RITA | Right internal thoracic artery |
| GCP | Good Clinical Practices |
| ECC | Extracorporeal circulation |
| IRB | Institutional review board |
| CNIL | French National Commission for Information Technology and Civil Liberties |
| CNRS | French National Centre for Scientific Research |
| CRF | Case Report Form |
| CVTS | Cardiovascular and thoracic surgery |
| ICH | International Conference on Harmonisation |
| RN | Registered nurse |
| INSERM | French National health and medical research institute |
| IPITA | Impact of Pulsatility on Internal Thoracic Arteries |
| PRES LUNAM | Research and Higher Education Centre of Angers University Nantes Le Mans University |
| SPC | Summary of product characteristics |
| CST | Clinical study technician |
| MRU | Mixed research unit |
|  |  |

# General information

## Roles

| Administrator | CHU d’Angers (Angers University Hospital)  4 rue Larrey  49 933 Angers Cedex 09  🕿 +3302 41 35 36 37 |
| --- | --- |
| Head of Research on behalf of the administrator | Ms. Elsa Livonnet,  Director of the Department of Medical Affairs and Research  CHU d’Angers (Angers University Hospital)  🕿 +3302 41 35 32 85 🖷 +3302 41 35 32 89  [Elsa.Livonnet@chu-angers.fr](mailto:Elsa.Livonnet@chu-angers.fr) |
| Coordination of research on behalf of the administrator | Ms. Denise Jolivot, Ms. Sybille Lazareff,  Promotion and Management Unit  CHU d’Angers (Angers University Hospital)  🕿 +3302 41 35 58 08 🖷 +3302 41 35 59 68  [DeJolivot@chu-angers.fr](mailto:DeJolivot@chu-angers.fr) |
| Pharmacy | Dr. Valérie Daniel, Dr. Astrid Darsonval  Experimental Products Unit-Vigilance  CHU d’Angers (Angers University Hospital)  🕿 +3302 41 35 35 44 🖷 +3302 41 35 46 57  [VaDaniel@chu-angers.fr](mailto:VaDaniel@chu-angers.fr) |
| Methodology | Dr. Elsa Parot-Schinkel  Methodology and Biostatistics Unit:  CHU Angers  🕿 +3302 41 35 58 55  Elparot@chu-angers.fr |
| Biostatistics: | Prof. Christophe Baufreton  Cardiac Surgery Department  🕿 +3302 41 35 45 73  chbaufreton@chu-angers.fr |
| Data Manager | Mr. Jean-Marie Chretien  Methodology and Biostatistics Unit:  CHU Angers  🕿 +3302 41 35 59 76  jmchretien@chu-angers.fr |

## Researchers

### Research Coordinator

Dr Olivier Fouquet, Unit UMR CNRS 6214-INSERM 1083, CCVT, CHU Angers

### Associate researchers

Associated investigators researchers and their contact details are detailed in an attached document titled "List of Associated Researchers".

## Scientific steering committee

- Associate researchers

Dr. Daniel Henrion, head of unit manager UMR CNRS 6214-INSERM 1083 unit

Dr. Laurent Loufrani, unit UMR CNRS 6214-INSERM 1083 unit

Dr. Frédéric Pinaud, Unit UMR CNRS 6214-INSERM 1083, CCVTCVTS, CHU Angers unit

CCVT CVTS perfusionist team, CHU Angers: Emmanuelle Bouquet, Laurence Verron, Antoine Marcesche, Anthony Fribault, Sébastien Girardot.

Emilie Dalmayrac, Ingénieur Attachée de Recherche CliniqueEngineer Attached to Clinical Research, CCVTCVTS, CHU Angers

Elise Houssin, Clinical Studies Technician, CCVTCVTS, CHU Angers

- Scientific committee

Dr. Daniel Henrion, unit managerhead of UMR CNRS 6214-INSERM 1083 unit

Prof. Christophe Baufreton, PRES LUNAM, Université d’AngersUniversity of Angers, UPRES EA 3860, CCVT, CHU Angers

# Scientific justification and general description of the research

## Name and description of the disease

Atherosclerosis causes progressive obstruction of the arteries and sometimes acute thrombotic complications. Myocardial infarction (MI) is a systemised ischaemic necrosis of the cardiac muscle, there are 120,000 cases in France and it is still responsible for 10 to 12 % of adult deaths every year. An estimated 13,528 surgical coronary revascularisation or coronary bypass procedures were carried out between 01/01/2010 and 31/10/2010 according to the primary Epicard database of the SFCTCV (French Society of Vascular and Thoracic Surgery). The average number of vascularised arteries was 2.91 per patient. In 2011, according to the same database, 58.7 % of procedures were associated with the use of 2 internal thoracic arterial grafts (ITA), 35.4 % with only one ITA and 5.8 % with saphenous bypass grafts only. The use of 2 ITA grafts affects concerns less than 4 % of revascularized patients in the USA (1). Loop et al. have demonstrated superiority of left ITAs on the anterior interventricular artery in long-term survival compared with saphenous grafts (2).

In routine practice, no consensus has been established on the choice of which arterial pump to use. Therefore, each centre, according to its own habits, uses either a roller pump and/or a centrifugal pump as an arterial infusion pump in ECC.

## Name and description of studied devices/strategies/procedures

This project concerns two types of arterial infusion pumps routinely used in ECC:

- **So-called occlusive roller pumps**: the most commonly used, these work on the principle of more or less complete occlusion of a flexible silicon tube by rotating rollers. The flow rate depends on the diameter of the tube (in general 1/2 inch in an adult), of the circumference of the support and the number of turns/minute of the pump (50-150 rpm). The rollers are adjusted in order to obtain a subocclusion which prevents the formed elements from being crushed but is sufficient to propel the blood mass. The roller pumps are independent of the afterload: they maintain their flow rate whatever the arterial pressure.

- **So-called non-occlusive centrifugal pumps**: These propel the blood via a rotating turbine. The difference of pressure between the centre and periphery created by the interior centrifugal force of the cone accelerates the blood which is ejected out. This pump doesn’t disturb the formed elements much and reduces platelet stimulation and haemolysis, but it is sensitive to the preload and afterload because it is not occlusive: an increase in arterial resistance reduces its flow rate.

The devices studied are detailed in chapter 6.

## Summary of the results of clinical and non-clinical trials available and relevant to the routine care study in question

Arterial grafts of internal thoracic arteries (ITA) are the best graft in terms of permeability in the long-term. These muscular or resistance arteries make up a large part of the vascular tree. These small arteries contain a large amount of smooth muscle cells in comparison to elastic arteries (thicker arteries) and play an important role in the systemic regulation of arterial pressure, for optimal distribution of the blood at tissue level. Numerous studies have compared the permeability of the grafts: 98 % at 5 years, 95 % at 10 years and 88 % at 15 years for left internal thoracic arteries (9). In this study Tatoulis et al. (9) demonstrated that the permeability of the graft depends on the type of conduit duct used, their distribution and the degree of coronary stenosis downstream. Other arterial grafts are also used such as radial arteries. However, unlike the internal thoracic arteries taken in situ, the radial arteries are like the saphenous veins, used as autografts, subjected to a tissue ischaemia-reperfusion sequence related to the procedure including the loss of the pulsatile flow in the absence of flow and pressure rate in the arterial lumen. Interestingly, radial arteries like the saphenous veins are prone to intimal hyperplasia (10). Comparative clinical studies have demonstrated no proven superiority of radial arteries over saphenous veins in terms of post-operative permeability after the surgery (10, 11). Regarding use of arterial or venous grafts as an autograft for coronary bypass, there has been little data on the impact of pulsatility or its absence, on endothelial structure or function.

In 2010, 87.8 % of coronary revascularisation procedures were performed under extra-corporeal circulation (ECC) according to the Epicard database. The arterial infusion pumps in ECC are:

Occlusive: still the most widely used, these are comprised of 2 rollers which turn around the PVC pipe creating a flow by compression of the blood

- Non-occlusive: by stretching or centrifugation, they cause less haemolysis and come from mechanical assistance.

The roller pumps produce a pulsatile flow rate (12) and can be adjusted to either boost or not boost the pulsed flow. The pulsatility of a flow rate depends more on the energy gradient than pressure gradient (13). During the operation, the use of a centrifugal pump generating a non-pulsatile flow rate is associated with an increase in inflammatory response (complement and neutrophil activation) for reasons that have yet to be fully understood (14). A recent study on an in-vitro model revealed that the loss of pulsatility in a mesenteric rat artery (resistance artery), independently of flow rate or pressure, generating a parietal inflammatory response after 30 minutes linked to oxidative stress, may be cancelled out by using an antioxidant (tempol) (15). The presence of pulsatility, in this in vitro study, causes a decrease in myogenic tone and early flow-mediated dilation. In the absence of pulsatility, any change in the pharmacological reactivity has not been seen and the non-pulsatile pressure leads to a significant production of inflammatory proteins and ROS. During an ECC for coronary bypass, the operating phase during which pulsatility can be eliminated is consistent with that of the aortic clamping when native cardiac activity has stopped due to cardioplegia. Residual pulsatility in the vascular system during aortic clamping is therefore solely dependent on the arterial pump used. Watarida et al have shown in patients undergoing coronary artery bypass grafting that the elevation of circulating concentrations of circulating endotoxin correlated with the duration of clamping in the presence of the non-pulsatile ECC (16). In the literature review published by Alghamdi (17), the only controlled and randomised trial deemed to be of good quality concluded that a pulsatile infusion (roller pump) during ECC was associated with a reduction of myocardial infarction, mortality and major complications (18). However, centrifugal pumps which deliver a non-pulsatile flow, still remain the most commonly used.

Thus, it seems essential to be able to assess the impact of pulsatility on arterial grafts in order to determine if a centrifugal pump (non-pulsatile) can have a negative impact on the mechanic function of these grafts that may explain some early occlusions.

## Justification for the qualification of the routine care study

The general objective of this routine care project is to assess and compare the impact of two types of arterial infusion pumps routinely used in ECC (roller and centrifugal pumps) on the endothelial function of ITAs:

1. In 2012, the cardiac surgery department at CHU d’Angers performed 637 major cardiac surgeries. In the same year, 202 patients underwent coronary bypass surgery alone with ITAs being used for more than 98 % of them. 92 % of the procedures were performed under ECC. The department is provided with 50 centrifugal pumps every year, the use of these pumps being selected randomly allowing the technique to become a habit for the cardiac perfusionists. The use of these pumps is random for both coronary surgery and valve surgery.
2. The only investigations specific to the study are blood samples in the unit as well as the study of tissue samples (segments of thoracic arteries usually considered to be surgical waste). Therefore, there is no specific risk or constraint for the patient in this study.
3. Current knowledge of the impact of a centrifugal or roller pump does not allow us to consider one technique to be superior to the other. Neither one has shown its superiority in terms of benefit and safety for the patient. However, numerous studies (14, 16, 17, 18) have shown that use of a roller pump significantly reduces the general inflammatory response. The only experimental study carried out by Pinaud et al. (15) has revealed a significant change in arterial vascular reactivity (mesenteric rat arteries) in the absence of a pulsatile flow. There is no data on the types of pumps used in France, only a telephone survey across 58 French cardiac surgery centres (out of 65) revealed that a centrifugal pump was used in 34% (n=20) of cases.

## Description of the study group

Male patients requiring surgical revascularisation with at least one ITA will be included in this study.

# Hypotheses and research objectives

## Hypotheses

The hypothesis of the I.P.I.T.A. protocol is that the use of a roller pump delivering a microcirculation pulsatility during the aortic clamping, will have a more beneficial impact on the endothelial function of the ITAs than the use of a centrifugal pump as it causes a change in myogenic tone in the internal thoracic artery (hypothesis developed due to the conclusions of Pinaud et al.’s experimental study).

If our hypothesis is proved to be correct, it would seem justified to change our surgical habits, meaning the systematic use of roller pumps after coronary bypass surgery.

## Objectives and judgement criteria

The objectives will be to assess according to the types of pumps used:

- the vascular reactivity of the internal thoracic artery;
- the oxidative stress and inflammatory response at wall level of the graft;
- the inflammatory response in the patient’s blood

The corresponding judgement criteria are as follows:

- percentage of contraction in terms of pressure (myogenic tone) and the percentage of dilation in terms of flow (vasodilatation) as well contraction and dilation phenomena in terms of pharmacological additions;
- ROS measurement (reactive oxygen species), histology assessment, MCP-1 (Monocyte chemotactic protein-1), TNF-α;
- measurement of the sC5b-9 complement chain and leukocyte elastase.

# Research design

## Justification for the planned number of people to include in the study

There is no data available in the literature to allow us to carry out a population calculation for this exploratory physiopathology study. The only article close in terms of methodology and objectives (15) concerns an experimental study on the mesenteric arteries of rats, carried out with 10 rats per group with a hundred rats in total.

A number of 40 patients per group (the ‘roller pump’ group or the ‘centrifugal pump’ group) is the standard commonly used in this type of study. Each patient will have 2 tissue samples taken: 1 before clamping and 1 during clamping just before anastomosis. Each tissue sample will be used to assess the vascular reactivity, biochemistry and histology.

## Description of measures taken to reduce and avoid bias

### Random selection

Product allocation will be carried by balanced randomisation. The randomisation procedure is carried out by the Department of Methodology and Biostatistics at CHU d’Angers (Angers University Hospital). The safety standards provided in Angers, for any surgery under ECC stipulate that the pump be installed on arrival of the patient in the operating room.

Therefore, the patients will be randomised the day before the surgery so that the preparation of the equipment necessary for the operation can be planned, the infusion pump for the ECC (roller or centrifugal pump) selected in accordance with the randomisation.

### Unblinding procedures

This is a single-blind study as the surgeon and cardiac perfusionists will be aware of which pump is installed in the operating room. Therefore, only the patient is unaware of the pump choice.

However, the blood and tissue samples will be anonymous, the laboratory engineers carrying out the different tests and experiments will not know which type of pump has been used so the assessment of the judgment criteria of the study will be done blind.

## Description of the research methodology, accompanied by its schematic presentation specifying in particular the visits and examinations

### Experimental plan

It is a study in routine care, monocentric, comparative, prospective, single blind, randomised.

This study concerns patients undergoing coronary bypass surgery at CHU d’Angers, in whom segments of internal thoracic artery necessary for bypass grafting as well as biological samples will be analysed in order to study the physiopathological impact of the type of arterial pump used in the course of ECC.

### Conduct of the study

#### Collection of the non-opposition/ Inclusion

The preselection of patients will be done in a medical-surgical meeting, from their medical record and their previous consultation, according to the various assessable selection criteria at that date and without notifying the subject.

During the inclusion visit, at the time of the surgical consultation, if the subject meets the study selection criteria, a notice of non-opposition will be collected by the researcher after an interview and the issuance of the information letter written in language understandable to the subject. The information letter is to be signed in at least two copies by the different parties.

#### Follow up of the subjects taking part in the research

During the visit carried out the day before the operation (D-1), a clinical examination (see chapter 7.1.2) will be carried out to verify the absence of recent operative contraindications (infections, destabilisation of coronary diseases, haemostasis problems, etc.) and to randomise the patient.

During the operation (D0), data relating to the operation as well as tissue and biological samples will be collected.


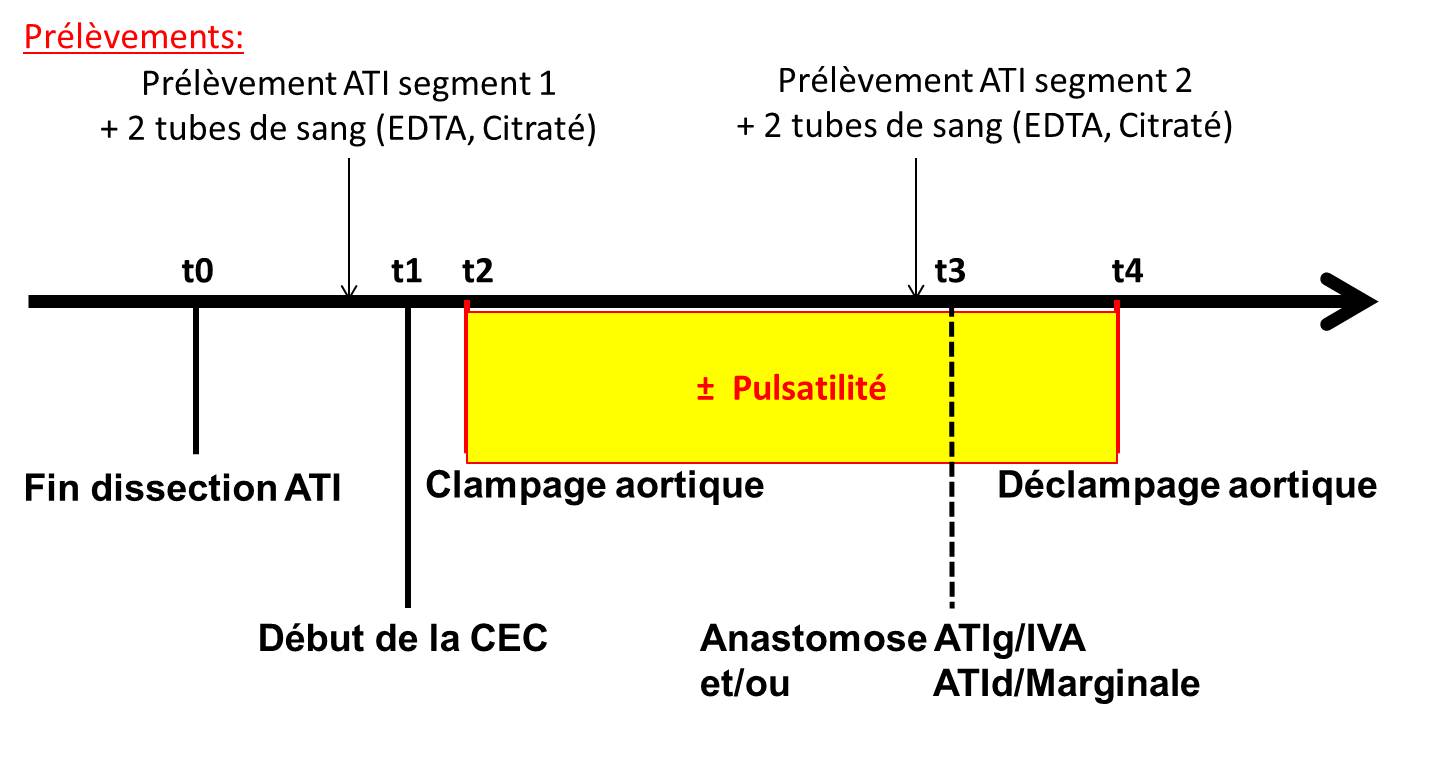


During coronary bypasses, the entire pedunculated internal thoracic artery (ITA), (i.e. attached to its proximal portion at the subclavian artery) will be removed and will measure around 25 to 30 cm in length. In order to adapt the adequate length to the patient, a segment of around 5 cm will be systematically cut, in the distal portion, this surplus is therefore operative waste.

This artery surplus will be the basis for the ITA tissue study.

The ITA segments will be removed in two stages:

- Just before clamping (an artery which will then be divided into 3 parts depending on the size of the surplus graft: 5 mm for immunohistochemistry, 5 mm for biochemistry and 5 mm for vascular reactivity).
- Just before the anastomosis (an artery which will then be divided into 3 parts depending on the size of the surplus graft)

Furthermore, 4 tubes of blood (2 EDTA, 2 citrated) will be taken during the operation as part of the protocol. The samples will be taken just before the removal of each ITA segment:

- 1 EDTA tube (3 ml), 1 citrated tube (4 ml) just before the sampling of the first ITA segment
- 1 EDTA tube, 1 citrated tube just before the sampling of the second ITA segment

In total, 14 ml of blood will be taken as part of this study.

The study does not provide for clinical follow up for patients after the operation. It would be impossible to remotely determine the clinical impact of the choice of the pump on such a small number of subjects.

## Expected duration of participation, chronology description, and the duration of all trial periods, including follow up, where necessary

Number of patients expected: 80

Inclusion period duration: 2 years

Period between inclusion and surgery: usual time for this type of operation (around 1 month)

Duration of participation in the study for a patient: Time under ECC during the coronary surgery. (+/- 1 hour)

Total duration of the study (inclusion duration + participation duration) : 2 years and 1 month

## Description of definitive or temporary stopping rules

### End of research participation for an individual

Patients can request to end their participation in the study at any time and for any reason.

The researcher can definitively stop a patient from participating in the study for any reason that serves the best interests of the patient.

Patient exclusion criteria is as follows:

- Patient refusing to the use of their data

- Contraindication to surgery or cancellation of the expected surgery

- Too short a graft length possibly compromising the bypass quality

- Precarious haemodynamic and/or haemorrhagic setting, making the sampling time unsuitable.

Patients leaving the study before randomisation will be replaced (inclusion and randomisation of 80 patients).

A patient leaving the study will not change anything regarding their routine care for their illness.

In the case that a patient leaves the study early and does not object to the use of their data, the researcher must document the reasons as clearly as possible.

The data recorded for subjects who have left the study early will be used for analysis unless the patient refuses the use of their data.

### Stopping a part of or the entirety of the research

Unforeseen events, where the objectives of the study or clinical programme are not likely to be reached, may cause the administrator to prematurely stop the study.

Angers University Hospital reserves the right to stop the study, at any time, if it deems that the inclusion goals have not been met.

In the case of a premature end to the study, information will be transferred to the IRB by the administrator within 15 days. In the case of a premature end to the study, the researchers must quickly inform the patients participating in the study.

# Selection and exclusion of patients for research

## Inclusion criteria of people taking part in the research

> 18 years,

Male

Elective surgery for a coronary bypass using at least one internal thoracic artery

These patients are volunteers and are not opposed to the use of their data

## Exclusion criteria for people taking part in the research

Female (due to the hormonal impact (oestrogen) on arterial vascular reactivity.

Combined surgery

Emergency surgery

Participation in another protocol

Opposing to the use of their data

## Mode of recruitment

Patients with planned surgery in the Cardiac Surgery department as part of their disease will be offered to take part in the study.

# Care and treatments administered to patients taking part in the research

## Description of studied devices/strategies/procedures

In standard practice, the pump choice is random. Cardioperfusionists use centrifugal or roller pumps at their convenience to ensure a "habit of use". The ECC circuit will be identical irrespective of the pump used: Sorin circuit ® Reference AB0395.

### Device/strategy/procedure A

Studied device A is the Maquet roller pump whose mechanism consists of a rotor with 2 rollers compressing a silicone tube.

### Device/strategy/procedure B

Studied device B is the Sorin centrifugal pump whose mechanism consists of a magnet causing the stretching of the blood, this technique being derived from the mechanical assistance

## Other treatments, devices, associated strategy(ies)

There will be no changes to patients' care.

# Evaluation criteria

## Evaluation criteria non specific to the study

### Inclusion visit

Patient clinical data will concern: age, sex, weight, height, dyslipidaemia, arterial hypertension, smoker, diabetes type 1 and 2, family cardiovascular history, atherosclerosis of subclavian arteries, anti-platelets, associated inflammatory diseases.

### Day before operating

Pre-operation clinical examination data will be recorded as well as, if needs be, the exclusion criteria (refusal, contraindications to surgery, etc.)

For pre-operation biological data: CRP, leukocytes

### Surgery

Said surgical data will include: the type of pump used, the type of circuit used, the anticoagulation level (measurement of target activated clotting time [ACT] based on Hepcon® HMS in the operating room and the dose of heparin injected).

## Evaluation criteria specific to the study

This evaluation criteria comes from the results of biological blood and tissue analysis. No other further follow up procedure will be offered as part of this study

### Tissue analysis

Thoracic artery segments necessary for the biochemical study, immunohistochemicals will be frozen using liquid nitrogen (-80°C) in the operating room, in a dedicated room, by a laboratory technician. The segments for the vascular reactivity study are kept fresh and forwarded with the other samples to the UMR CNRS 6214-INSERM 1083 laboratory by the technician.

Analyses carried out from these samples will include:

1. Analysis of arterial vascular reactivity: percentage of dilation and contraction depending on the pulsatility and pharmacology. The arteries, being isolate and fresh, will be mounted on an arteriography and infused with physiological saline without circulating cells.

- Evaluation of the myogenic tonicity: measurement of the passive diameter of the artery when it is subjected to different pressure regimes (10, 25, 50, 75, 100, 125 and 150 mmHg) according to the time.
- Evaluation of vasodilatation (dilatation dependent flux): measurement of the diameter of the artery depending on the flow circulating in the artery which is increased step by step from 0 to 100 μl / min according to the time.
- Study of the vascular reactivity according to the adding of pharmacological agents: antioxidant (TEMPOL)

1. Analysis of parietal oxidative stress: ROS measurement (reactive oxygen species)
2. Histology analysis
3. Immunohistochemical analysis: MCP-1 (Monocyte chemotactic protein-1), TNF-α

### Blood analyses

Four tubes of blood will be taken just before the sampling of each graft segment:

- 2 EDTA tubes (3 ml of blood/tube) for the study of the complement chain: sC5b-9

- 2 citrated tubes (4 ml of blood/tube) for the study of leukocyte elastase

# Safety assessment

In the case of a routine care study, since the procedures are carried out in accordance with usual practices, the declaration of adverse effects related to the care is part of the hospital centre's usual vigilance channel where the research is conducted: pharmacovigilance if the effect involves a drug, materiovigilance for a medical device, risk management, etc.

## Description of the parameters for assessing safety

Regarding the monitoring-specific examinations within the study protocol, there is no need to provide the secondary adverse event.

The tubes of blood will be taken using perfusions previously used for other analyses, the samples will be taken in the operating room under general anaesthetic.

## Safety Monitoring Committee

In view of the nature of the study and absence of unexpected adverse effects related to the specific research of the study, no safety monitoring committee has been established.

# Summary of benefits, if any, and foreseeable risks known for those suitable for research

## Benefits

### Individual benefits

There is no expected individual benefits taking into account the fact that it is a study into routine care.

### Collective benefits

This study will allow for a better understanding of the mechanisms of deterioration of mammary grafts that may lead to the resurgence of angina pectoris, infarction. If it is proved that the absence of a pulsating flow during the ECC leads to a significant alteration of the grafts' lining, the systematic use of a roller pump for coronary bypasses must be considered.

## Risks

No individual or collective risks are expected in this routine care study.

In practical terms, the tissue samples are operative waste.

The tubes of blood will be taken using perfusions previously used for other analyses, the samples will be taken in the operating room under general anaesthetic.

## Balance benefits / risks

The risks and the predictable constraints for the patients participating in the study, described in paragraphs above are minimal with regard to the expected benefits.

# Data management and treatment

## Case report form

The case report form is currently being created and will contain the data detailed below.

All information required by the protocol must be recorded in the case report form and an explanation must be given for any missing information. Data must be collected as and when it is obtained, and neatly recorded in the case report form so as to be legible.

Any incorrect data found in the notebooks will be clearly crossed out and the new data will be copied, next to the crossed out information, accompanied by the initials, the date and possibly a justification by the researcher or the person authorised to make this correction.

## Data entry

The data will be entered by Doctor Olivier Fouquet, cardiac surgeon, thoracic and cardiovascular surgery on anonymised data files.

Data analysis will be carried out by Doctors Daniel Henrion, Laurent Loufrani and Olivier Fouquet.

## Archiving

The following documents will be archived by the promotion unit at Angers University Hospital and in its cardiac surgery department until the end of the period of practical use in accordance with current regulations.

These documents are as follows:

- Protocol and appendices, eventual amendments,
- Signed information letters (non-opposition)
- Individual data (authenticated copies of raw data)
- Documents relating to study products
- Statistical Analysis
- Final Study Report or abstract of the final report

At the end of the period of practical use, all documents to be archived will be placed under the responsibility of the administrator and the principal researchers for 15 years after the end of the study, in accordance with institutional practices.

No data can be destroyed without agreement from the administrator. After 15 years, the administrator will be consulted to destroy data. All data, documents and reports may be subject to audit or inspection.

# Quality control and assurance

The principal researcher will ensure the successful execution of the study, data collection, the documentation, recording and reporting, adhering to the Standard Operating Procedures that apply throughout Angers University Hospital and in accordance with Good Clinical Practices as well as current legal provisions and regulations

# Statistics

Statistical analysis will be carried out at the UMR CNRS 6214-INSERM 1083 unit (Dr Daniel Henrion) and in the cardiac surgery department (Prof. Christophe Baufreton).

Software used: SPSS V15 (Chicago, IL, USA)

## Description of intended statistical methods, including the schedule of planned intermediate analyses

The flowchart of patients (included or not) will be used as well as a descriptive analysis of the characteristics of the participants.

For qualitative variables, the results will be reported in numbers and percentages. For quantitative variables, the results will be reported using averages and standard deviation in the case of normal distribution and in median with a 25th and 75th percentile in the absence of Gaussian distribution for the considered variable.

For bivariate analysis, the use of parametric or non-parametric tests, will be based up on the number and/or the distribution of variables. The significance threshold was set at 0.05 and all tests will be bilateral.

The statistical test used will be the Chi-squared test by Pearson (or the Fisher exact test) for qualitative variables. For qualitative variables, the statistical tests used will be the Student t test (or the non-parametric test by Mann-Whitney) to compare two groups and one ANOVA (or the non-parametric test of Kruskall Wallis) to compare 3 or more groups.

## Expected degree of statistical significance

The significance threshold was set at 0.05 and all tests will be bilateral.

## Statistical criteria for discontinuation of study

Not applicable

## Missing, unused or invalid data inclusion method

No allocation method will be used in the case of missing data.

## Management of changes made to the initial strategy analysis plan

Statistical analysis will be carried out in accordance with the pre-established analysis plan.

## Choice of people to be included in the analysis.

All individuals for whom the evaluation criteria will be available will be included in the analysis.

# Right of access to data and source documents

## Access to data

The medical data of each patient will only be transferred to the agency attached to the person responsible for the research or any person duly authorised by the person responsible for the research under the conditions guaranteeing their confidentiality.

## Source documents

If need be, the organism attached to the responsible person may request direct access to the medical record to verify procedure and/or research data, without violating confidentiality and in the limits authorised by law and regulations.

# Ethical considerations and regulations

## Institutional Review Board

The study file (including the protocol, the list of associated researchers and the study information letter) will be submitted for approval to the Institutional Review Board.

From the first inclusion, the administrator must immediately inform the Institutional Review Board of the effective trial start date.

The trial end date shall be submitted to the Institutional Review Board within 90 days. The research end date corresponds to the end of participation of the last test subject, or, if applicable, to the end date defined by the protocol.

## Substantial modifications

In the case of substantial modifications being made by the researcher to the study file, they will be approved by the administrator. The administrator will have to obtain approval from the IRB before its implementation.

## National Commission for Information Technology and Civil Liberties

In accordance with law no. 78-17 of 6 January, 1978 relating to IT and Civil Liberties, modified by the law of 1 August, 1994 relating to the treatment of nominative data used for healthcare research and the law of 6 August, 2004, relating to the protection of physical persons and the treatment of their personal data, in terms of strictly mono-centric data treatment, only authorisation concerning the treatment of data necessary for the study will be requested to the National Commission for Information Technology and Civil Liberties.

## Data confidentiality

The person in charge of the research and those with direct access to the data will take all necessary precautions in order to ensure the confidentiality of the information relating to the appropriate persons, including their identity. These persons are bound by professional secrecy (according to the terms defined by articles 226-13 and 226-14 of the Penal Code). In this case, tissue and blood samples will be sent anonymously to the person in charge of the research without any information to identify the donor.

## Insurance

As the research is registered as Routine Care Research by the requested IRB, which means no additional risks are associated with the study, the insurance will be that of the institution responsible for care (Article L. 1142-2).

Angers University Hospital has taken out an insurance policy with SHAM (number 127049) for civil liability towards patients, to cover obligations under their responsibility for care.

# Feasibility of the study

Angers University Hospital's cardiac surgery department has carried out 637 major operations, and more than 10,000 extracorporeal circulations in 20 years with national recognition in extracorporeal circulation studies.

In 2012, outside of associated valvular acts, 202 patients had coronary bypasses, the number of anastomosis per patient being 2.5/patient. In more than 90 % of procedures carried out, an ITA was used and 92 % of operations will be carried out under ECC.

# Regulations relating to publication

Scientific communications and reports corresponding to this study will be done under the responsibility of the person in charge of the research with the approval of the associated researchers. The co-authors of the report and publications will be the clinicians involved, in proportion to their contribution to the study, as well as the biostatistician and associated researchers.

The rules of publications will follow the international recommendations (N Engl J Med 1997; 336: 309-315).

The study may be recorded on an open-access website (clinical trial) before the collection of the 1st patient's data.

# References to scientific literature and relevant data as a reference for research

1. Sabik JF. Understanding Saphenous Vein Graft Patency. Circulation. 2011Jul.18;124(3):273–5.

2. Loop FD, Lytle BW, Cosgrove DM, Stewart RW, Goormastic M, Williams GW, et al. Influence of the internal-mammary-artery graft on 10-year survival and other cardiac events. N Engl J Med. 1986Jan.2;314(1):1–6.

3. Sharma GV, Deupree RH, Khuri SF, Parisi AF, Luchi RJ, Scott SM. Coronary bypass surgery improves survival in high-risk unstable angina. Results of a Veterans Administration Cooperative study with an 8-year follow-up. Veterans Administration Unstable Angina Cooperative Study Group. Circulation. 1991Nov.;84(5 Suppl):III260–7.

4. Thatte H, Khuri S. The coronary artery bypass conduit: I. Intraoperative endothelial injury and its implication on graft patency. Ann Thorac Surg. 2001;72(6):S2245–52.

5. Lopes RD, Mehta RH, Hafley GE, Williams JB, Mack MJ, Peterson ED, et al. Relationship Between Vein Graft Failure and Subsequent Clinical Outcomes After Coronary Artery Bypass Surgery. Circulation. 2012Feb.13;125(6):749–56.

6. Rousou L, Taylor K, Lu X, Healey N, Crittenden M, Khuri S, et al. Saphenous vein conduits harvested by endoscopic technique exhibit structural and functional damage. Ann Thorac Surg. 2009;87(1):62–70.

7. Sun Q, Kawamura T, Masutani K, Peng X, Sun Q, Stolz DB, et al. Oral intake of hydrogen-rich water inhibits intimal hyperplasia in arterialized vein grafts in rats. Cardiovasc Res. 2012Apr.1;94(1):144–53.

8. Mehta RH, Ferguson TB, Lopes RD, Hafley GE, Mack MJ, Kouchoukos NT, et al. Saphenous Vein Grafts With Multiple Versus Single Distal Targets in Patients Undergoing Coronary Artery Bypass Surgery: One-Year Graft Failure and Five-Year Outcomes From the Project of Ex-Vivo Vein Graft Engineering via Transfection (PREVENT) IV Trial. Circulation. 2011Jul.18;124(3):280–8.

9. Tatoulis James, FRACS, Buxton F. Brian, FRACS, and Fuller John A, FRACP. Patencies of 2,127 Arterial Conduits over 15 Years. Ann Thorac Surg 2004;77:93-101.

10. Buxton BF, Hayward PAR, Newcomb AE, Moten S, Seevanayagam S, Gordon I. Choice of conduits for coronary artery bypass grafting: craft or science? European Journal of Cardio-Thoracic Surgery. 2009Apr.1;35(4):658–70.

11. Sun JCJ, Teoh KHT, Lamy A, Sheth T, Ellins ML, Jung H, et al. Randomized trial of aspirin and clopidogrel versus aspirin alone for the prevention of coronary artery bypass graft occlusion: the Preoperative Aspirin and Postoperative Antiplatelets in Coronary Artery Bypass Grafting study. Am Heart J. 2010Dec.1;160(6):1178–84.

12. James SA, Peters J, Maresca L, Kalush SL, Trigueros EA. The roller pump does produce pulsatile flow. J Extra Corpor Technol. 1987Oct.27;19:376–83.

13. Driessen JJ, Dhaese H, Fransen G, Verrelst P, Rondelez L, Gevaert L, et al. Pulsatile compared with nonpulsatile perfusion using a centrifugal pump for cardiopulmonary bypass during coronary artery bypass grafting. Effects on systemic haemodynamics, oxygenation, and inflammatory response parameters. Perfusion. 1995;10(1):3–12.

14. Baufreton C, Intrator L, Jansen PG, Velthuis te H, Le Besnerais P, Vonk A, et al. Inflammatory response to cardiopulmonary bypass using roller or centrifugal pumps. Ann Thorac Surg. 1999Apr.1;67(4):972–7.

15. Pinaud F, Loufrani L, Toutain B, Lambert D, Vandekerckhove L, Henrion D, et al. In vitro protection of vascular function from oxidative stress and inflammation by pulsatility in resistance arteries. J Thorac Cardiovasc Surg. 2011Nov.;142(5):1254–62.

16. Watarida S, Mori A, Onoe M, Tabata R, Shiraishi S, Sugita T, et al. A clinical study on the effects of pulsatile cardiopulmonary bypass on the blood endotoxin levels. J Thorac Cardiovasc Surg. 1994Oct.;108(4):620–5.

17. Alghamdi AA, Latter DA. Pulsatile versus nonpulsatile cardiopulmonary bypass flow: A evidence-based approach. J Card Surg. 2006;21:347-354.

18. Murkin JM, Martzke JS, Buchan AM, Bentley C, Wong CJ. A randomized study of the influence of perfusion technique and ph management strategy in 316 patients undergoing coronary artery bypass surgery. Neurologic and cognitive outcomes. The Journal of thoracic and cardiovascular surgery. 1995;110:349-362.
