# Supplementary material for: Cardiopulmonary bypass and internal thoracic artery: Can roller or centrifugal pumps change vascular reactivity of the graft? The IPITA study: A randomized controlled clinical trial
Source: PLoS One. 2020 Jul 9;15(7):e0235604. doi: 10.1371/journal.pone.0235604 (PMC7347139; doi:10.1371/journal.pone.0235604)
Supplement: S2 Appendix — (PDF) [file pone.0235604.s005.pdf]

CHU Angers – Registre CNIL

Traitements à finalité recherche ou réalisés dans le cadre de la recherche

**Traitement n° 2015-002**	**IPATI**
Date d'inscription dans le registre	27/01/2015
Type de déclaration	Déclaration normale
**Objectif de la recherche**	Analyse de l'impact de la pulsatilité sur la vasoréactivité artérielle et la réponse inflammatoire sur des artères thoraciques internes.
**Type de recherche**	soins-courant
	Mono centrique
**Date de mise en œuvre**	Janvier/2015
**Nombre de patients/personnes concernées**	80 patients
Récupération de données auprès du médecin traitant/spécialiste de ville/autre centre hospitalier impliqué dans le suivi	NON
Responsable du traitement et Service chargé de la mise en œuvre	Dr Olivier FOUQUET – Service de Chirurgie Cardiaque
Modalité d'information des patients/personnes sur le traitement réalisé	Lettre d'information avec traçabilité de la recherche de non-opposition
Fonction de la personne ou du service auprès duquel s'exerce le droit d'accès	Dr Olivier FOUQUET – Service de Chirurgie Cardiaque
Logiciels utilisés	Epidata/Excel
Mesures de contrôle d'accès	Données stockées dans un dossier en accès protégé sur la zone réseau réservée au service
**Données traitées**	**Détails**
Données d'identification	1ère lettre du nom, 1ère lettre du prénom, DDN
Données de santé	Données issues du dossier médical du patient : atcd, données biologiques, données liées à l'intervention,…
**Catégories de destinataires**	**Données concernées**
Aucun	Les données sont utilisées uniquement au sein du service et ne font pas l'objet d'une transmission à l'extérieur de l'établissement
**Durée de conservation**	
**Mises à jour (Date et objet)**	
Néant	
