# Supplementary material for: Cardiopulmonary bypass and internal thoracic artery: Can roller or centrifugal pumps change vascular reactivity of the graft? The IPITA study: A randomized controlled clinical trial
Source: PLoS One. 2020 Jul 9;15(7):e0235604. doi: 10.1371/journal.pone.0235604 (PMC7347139; doi:10.1371/journal.pone.0235604)
Supplement: S3 Appendix — (DOC) [file pone.0235604.s006.doc]

**Impact de la Pulsatilité sur les Artères Thoraciques Internes**

**IPATI**

Version n°6 – 09/09/2016

**Numéro EUDRACT /ID :** 2014-A01247-40

| **Gestionnaire** |  |
| --- | --- |
| CHU d’Angers  4 rue Larrey  49 933 Angers Cedex 09 | **🕿** 02 41 35 36 37 |
| Investigateur Principal | |
| Dr Olivier FOUQUET  Service Chirurgie cardiaque  CHU d’Angers | 🕿 02 41 35 34 78  olfouquet@chu-angers.fr |
| **Soin courant** |  |
| **DISPOSITIF/STRATEGIE/PROCEDURE**  **INDICATION(S) CIBLE(S)**  **Type, Objet et Nature de la recherche** | Pompes artérielles pour CEC (pompe centrifuge ou pompe à galets)  Patient opéré sous CEC pour un pontage coronaire électif avec utilisation d’au moins un greffon d’artère thoracique interne  Soins Courants, Dispositifs médicaux, Thérapeutique |

| **AUTORISATIONS** | | |
| --- | --- | --- |
| **Comité de Protection des Personnes** | | |
| CPP Ouest N°2 | Numéro dossier : | Date de l'avis favorable : 16/01/2015 |
| **Comité consultatif sur le traitement de l’information en matière de recherche dans le domaine de la santé** | | |
| Numéro dossier : 2014/29 | | Date de l'avis favorable : NA |
| **Commission Nationale Informatique et Liberté** | | |
| Numéro dossier : 2015/002 | | Date de l'avis favorable : 27/01/2015 |

CE DOCUMENT CONFIDENTIEL EST LA PROPRIETE DU CHU D’ANGERS. AUCUNE INFORMATION NON PUBLIEE FIGURANT DANS CE DOCUMENT NE PEUT ETRE DIVULGUEE SANS AUTORISATION ECRITE PREALABLE DU CHU D’ANGERS.

**Sommaire**

[1. Informations générales 8](#__RefHeading___Toc398812365)

[1.1. Promotion 8](#__RefHeading___Toc398812366)

[1.2. Investigateurs 9](#__RefHeading___Toc398812367)

[1.2.1. Investigateur coordonnateur 9](#__RefHeading___Toc398812368)

[1.2.2. Investigateurs associés 9](#__RefHeading___Toc398812369)

[1.3. Comité scientifique et de pilotage 9](#__RefHeading___Toc398812370)

[2. Justification scientifique et description générale de la recherche 9](#__RefHeading___Toc398812371)

[2.1. Dénomination et description de la maladie 9](#__RefHeading___Toc398812372)

[2.2. Dénomination et description des dispositifs / stratégies / procédures étudié(e)s 10](#__RefHeading___Toc398812373)

[2.3. Résumé des résultats des essais non cliniques et des essais cliniques disponibles et pertinents au regard de la recherche en soins courants concernée 10](#__RefHeading___Toc398812374)

[2.4. Justification de la qualification de la recherche en soins courants 11](#__RefHeading___Toc398812375)

[2.5. Description de la population à étudier 12](#__RefHeading___Toc398812376)

[3. Hypothèses et objectifs de la recherche 12](#__RefHeading___Toc398812377)

[3.1. Hypothèses 12](#__RefHeading___Toc398812378)

[3.2. Objectifs et critères de jugement 12](#__RefHeading___Toc398812379)

[4. Conception de la recherche 13](#__RefHeading___Toc398812380)

[4.1. Justification du nombre prévu de personnes à inclure dans la recherche 13](#__RefHeading___Toc398812381)

[4.2. Description des mesures prises pour réduire et éviter les biais 13](#__RefHeading___Toc398812382)

[4.2.1. Tirage au sort 13](#__RefHeading___Toc398812383)

[4.2.2. Méthodes de mise en insu 13](#__RefHeading___Toc398812384)

[4.3. Description de la méthodologie de la recherche, accompagnée de sa présentation schématique précisant notamment les visites et les examens prévus 14](#__RefHeading___Toc398812385)

[4.3.1. Plan expérimental 14](#__RefHeading___Toc398812386)

[4.3.2. Déroulement de l'étude 14](#__RefHeading___Toc398812387)

[4.3.2.1. Recueil de la non-opposition / Inclusion 14](#__RefHeading___Toc398812388)

[4.3.2.2. Suivi des personnes se prêtant à la recherche 14](#__RefHeading___Toc398812389)

[4.1. Durée prévue de participation des personnes et description de la chronologie et de la durée de toutes les périodes de l’essai, y compris le suivi, le cas échéant 15](#__RefHeading___Toc398812390)

[4.2. Description des règles d’arrêt définitif ou temporaire 16](#__RefHeading___Toc398812391)

[4.2.1. Arrêt de participation d’une personne à la recherche 16](#__RefHeading___Toc398812392)

[4.2.2. Arrêt d’une partie ou de la totalité de la recherche 16](#__RefHeading___Toc398812393)

[5. Sélection et exclusion des personnes de la recherche 17](#__RefHeading___Toc398812394)

[5.1. Critères d’inclusion des personnes qui se prêtent à la recherche 17](#__RefHeading___Toc398812395)

[5.2. Critères de non inclusion des personnes qui se prêtent à la recherche 17](#__RefHeading___Toc398812396)

[5.3. Modalités de recrutement 17](#__RefHeading___Toc398812397)

[6. Prise en charge et traitements administrés aux personnes qui se prêtent à la recherche 17](#__RefHeading___Toc398812398)

[6.1. Description des dispositifs / stratégies / procédures étudié(e)s 17](#__RefHeading___Toc398812399)

[6.1.1. Dispositif / stratégie / procédure A 17](#__RefHeading___Toc398812400)

[6.1.2. Dispositif / stratégie / procédure B 17](#__RefHeading___Toc398812401)

[6.2. Autres traitements, dispositifs, stratégies associé(e) s 17](#__RefHeading___Toc398812402)

[7. Critères d’évaluation 18](#__RefHeading___Toc398812403)

[7.1. Critères d’évaluation non spécifiques à l’étude 18](#__RefHeading___Toc398812404)

[7.1.1. Visite d’inclusion 18](#__RefHeading___Toc398812405)

[7.1.2. Veille de l’intervention 18](#__RefHeading___Toc398812406)

[7.1.3. Intervention 18](#__RefHeading___Toc398812407)

[7.2. Critères d’évaluation spécifiques à l’étude 18](#__RefHeading___Toc398812408)

[7.2.1. Analyses tissulaires 18](#__RefHeading___Toc398812409)

[7.2.2. Analyses sanguines 19](#__RefHeading___Toc398812410)

[8. Evaluation de la sécurité 19](#__RefHeading___Toc398812411)

[8.1. Description des paramètres d’évaluation de la sécurité 19](#__RefHeading___Toc398812412)

[8.2. Comité de Surveillance 19](#__RefHeading___Toc398812413)

[9. Résumé des bénéfices, le cas échéant, et des risques prévisibles et connus pour les personne se prêtant à la recherche 19](#__RefHeading___Toc398812414)

[9.1. Bénéfices 19](#__RefHeading___Toc398812415)

[9.1.1. Bénéfice individuel 19](#__RefHeading___Toc398812416)

[9.1.2. Bénéfice collectif 19](#__RefHeading___Toc398812417)

[9.2. Risques 20](#__RefHeading___Toc398812418)

[9.3. Balance bénéfices / risques 20](#__RefHeading___Toc398812419)

[10. Traitement des données et gestion des données 20](#__RefHeading___Toc398812420)

[10.1. Cahier d’observation 20](#__RefHeading___Toc398812421)

[10.2. Saisie des données 20](#__RefHeading___Toc398812422)

[10.3. Archivage 20](#__RefHeading___Toc398812423)

[11. Contrôle et assurance de la qualité 21](#__RefHeading___Toc398812424)

[12. Statistiques 21](#__RefHeading___Toc398812425)

[12.1. Description des méthodes statistiques prévues, y compris du calendrier des analyses intermédiaires prévues 21](#__RefHeading___Toc398812426)

[12.2. Degré de signification statistique prévu 21](#__RefHeading___Toc398812427)

[12.3. Critères statistiques d’arrêt de la recherche 21](#__RefHeading___Toc398812428)

[12.4. Méthode de prise en compte des données manquantes, inutilisées ou non valides 22](#__RefHeading___Toc398812429)

[12.5. Gestion des modifications apportées au plan d’analyse de la stratégie initiale 22](#__RefHeading___Toc398812430)

[12.6. Choix des personnes à inclure dans les analyses 22](#__RefHeading___Toc398812431)

[13. Droit d’accès aux données et documents source 22](#__RefHeading___Toc398812432)

[13.1. Accès aux données 22](#__RefHeading___Toc398812433)

[13.2. Documents source 22](#__RefHeading___Toc398812434)

[14. Considérations éthiques et réglementaires 22](#__RefHeading___Toc398812435)

[14.1. Comité de Protection des Personnes et Autorité compétente 22](#__RefHeading___Toc398812436)

[14.2. Modifications substantielles 22](#__RefHeading___Toc398812437)

[14.3. CNIL 23](#__RefHeading___Toc398812438)

[14.4. Confidentialité des données 23](#__RefHeading___Toc398812439)

[14.5. Assurance 23](#__RefHeading___Toc398812440)

[15. Faisabilité de l'étude 23](#__RefHeading___Toc398812441)

[16. Règles relatives à la publication 24](#__RefHeading___Toc398812442)

[17. Références à la littérature scientifique et aux données pertinentes servant de référence pour la recherche 24](#__RefHeading___Toc398812443)

[18. Liste des annexes 25](#__RefHeading___Toc398812444)

SIGNATURES

| J'ai lu l’ensemble des pages du protocole de l’étude en soins courants dont le CHU d'Angers est le gestionnaire. Je m'engage à réaliser l’étude en respectant le protocole et les termes et conditions qui y sont définis. Je m'engage à réaliser l’essai en respectant :   - les règles et recommandations de bonnes pratiques cliniques françaises - les dispositions du Code de la Santé Publique relatives aux Recherches en Soins Courants,   Je m'engage également à ce que les investigateurs et les autres membres qualifiés de mon équipe aient accès aux copies de ce protocole et des documents relatifs à la conduite de l’étude pour leur permettre de travailler dans le respect des dispositions figurant dans ces documents. | |
| --- | --- |
| SIGNATURE DE L'INVESTIGATEUR | |
| **NOM: Dr Olivier FOUQUET**  Signature : …………………………………………….. | Date : ___________________ |

| SIGNATURE DU GESTIONNAIRE | |
| --- | --- |
| **NOM** : _______________________________________  Signature : ……………………………………………… | Date : ___________________ |

**Résumé**

| Titre | **Protocole IPATI** - Analyse de l’impact de la pulsatilité sur la vasoréactivité artérielle et la réponse inflammatoire sur des artères thoraciques internes |
| --- | --- |
| Gestionnaire | CHU Angers |
| Investigateur Coordinateur | Dr Olivier FOUQUET  Service chirurgie cardiaque  CHU Angers, 49933 Angers cedex 09 |
| Version du protocole | ***Version n°6 –*** |
| Justification / contexte | Les pathologies cardio-vasculaires sont la première cause de mortalité et le vieillissement de la population en accroît l’importance. La revascularisation coronaire chirurgicale est estimée à presque 15000 procédures par an. La grande majorité des patients auront une artère thoracique interne comme greffon utilisé pour revasculariser une des principales artères coronaires. L’utilisation d’une circulation extra-corporelle concerne 90% des procédures et impose un débit pulsé ou non pulsé en fonction du type de pompe artérielle. L’absence de pulsatilité entraîne une augmentation de la réaction inflammatoire, une altération du tonus myogénique dans la microcirculation comme cela a été montré dans des études expérimentales. Nous ne savons pas encore quel est l’impact de l’absence ou non de pulsatilité sur les greffons artériels et l’incidence que cela peut avoir sur leur perméabilité. |
| Objectif General | Evaluer et comparer l’impact sur la fonctionnalité endothéliale des ATI nécessaires à la chirurgie pour pontage coronaire de 2 types de pompes utilisés en pratique courante pour la CEC (pompes à galets et pompes centrifuges) |
| Objectifs | - Etude la vasoréactivité de l’artère thoracique interne en fonction du type de pompe utilisée (pulsatile ou non pulsatile) - Etude de la réaction inflammatoire pariétale en fonction de la pulsatilité - Etude de la réaction inflammatoire générale en fonction de la pulsatilité |
| Critère de Jugement | Etude de la vasoréactivité de l’artère thoracique interne :   - Vasodilatation (dilatation flux dépendant): Pourcentage de dilatation en fonction du débit - Tonus myogénique : Pourcentage de contraction en fonction de la pression   Mesure du stress oxydatif pariétal et systémique |
| Méthodologie / Schéma de l’etude | Recherche clinique interventionnelle en soins courants, prospective, randomisée, monocentrique, visant à évaluer deux dispositifs médicaux de classe IIb.  L’étude, réalisée au cours d’une CEC avec une pompe centrifuge (débit non pulsé) ou une pompe à galets (débit pulsé), compare l’impact sur la viabilité et fonctionnalité endothéliale des greffons artères thoraciques internes.  Deux groupes de patients : groupe 1 (pompe centrifuge), groupe 2 (pompe à galets).  Dans chaque groupe, prélèvements de segments d’artères avant l’anastomose coronaire :   - Avant la mise en CEC : l’artère est soumise au débit cardiaque - Pendant le clampage aortique, avant l’anastomose : l’artère est soumise au débit de la pompe artérielle   Analyses réalisées sur les segments artériels : vasoréactivité, mécanotransduction, histologie, immunohistochimie, biochimie  Dans chaque groupe, des prélèvements sanguins seront réalisés avant le début de la CEC et pendant le clampage, dosages réalisés juste avant le prélèvement du segment artériel :   - Sc5b-9 - Elastase leucocytaire |
| Critères d’Inclusion des patients | Patients âgés de plus de 18 ans  Homme  Pontage coronaire électif avec utilisation d’au moins une artère thoracique interne |
| Critères de Non-Inclusion des patients | Chirurgie en urgence  Femme  Chirurgie combinée  Participation à un autre protocole |
| Dispositifs / Stratégies / Procédures | En pratique courante, le choix de la pompe, centrifuge ou à galets, est déterminé de façon aléatoire.  Dans le cadre de l’étude, le type de pompe utilisé dépendra de la randomisation, la technique chirurgicale ne sera pas modifiée. |
| Nombre de patients | 80 patients |
| Durée de la Recherche | Durée de la période d’inclusion : 2 ans  Durée de l’étude pour un patient : environ 1 mois  Durée totale de l’étude : 2 ans et 1 mois |
| Retombées attendues | Evaluer l’intérêt de maintenir un débit pulsé pendant la CEC en termes de détérioration du tonus myogénique et d’altération des cellules endothéliales des ATI.  Apporter des arguments complémentaires en faveur d’une recommandation de l’utilisation des pompes à galets au cours de la chirurgie pour pontages coronaires. |

**Liste des abréviations**

| AMM | Autorisation de Mise sur le Marché |
| --- | --- |
| ARC | Attaché de Recherche Clinique |
| ATIg | Artère thoracique interne gauche |
| ATId | Artère thoracique interne droite |
| BPC | Bonnes Pratiques Cliniques |
| CEC | Circulation Extra-Corporelle |
| CPP | Comité de Protection des Personnes |
| CNIL | Commission Nationale de l’Informatique et des Libertés |
| CNRS | Centre National de la Recherche Scientifique |
| CRF | Case Report Form (cahier d’observation) |
| CCVT | Chirurgie CardioVasculaire et Thoracique |
| ICH | International Conference on Harmonization (Conférence internationale pour l'harmonisation) |
| IDE | Infirmière Diplômée d'Etat |
| INSERM | Institut National de la Santé et de la Recherche Médicale |
| IPATI | Impact de la Pulsatilité sur les Artères Thoraciques Internes |
| PRES LUNAM | Pôle de Recherche et d’Enseignement Supérieur de L’Université Angers Nantes Le Mans |
| RCP | Résumé des Caractéristiques d'un Produit |
| TEC | Technicien d'Etude Clinique |
| UMR | Unité Mixte de Recherche |
|  |  |

# Informations générales

## Promotion

| Gestionnaire | CHU d’Angers  4 rue Larrey  49 933 Angers Cedex 09  🕿 02 41 35 36 37 |
| --- | --- |
| Responsable de la recherche au nom du gestionnaire | Mme Elsa LIVONNET,  Directrice de la Direction des Affaires Médicales et de la Recherche  CHU d’Angers  🕿 02 41 35 32 85 🖷 02 41 35 32 89  [Elsa.Livonnet@chu-angers.fr](mailto:Elsa.Livonnet@chu-angers.fr) |
| Coordination de la recherche au nom du gestionnaire | Mme Denise JOLIVOT, Mme Sybille LAZAREFF,  Cellule de Promotion et Gestion  CHU d’Angers  🕿 02 41 35 58 08 🖷 02 41 35 59 68  [DeJolivot@chu-angers.fr](mailto:DeJolivot@chu-angers.fr) |
| Pharmacie | Dr Valérie DANIEL, Dr Astrid DARSONVAL  Cellule Produits Expérimentaux-Vigilance  CHU d'Angers  🕿 02 41 35 35 44 🖷 02 41 35 46 57  [VaDaniel@chu-angers.fr](mailto:VaDaniel@chu-angers.fr) |
| Méthodologie | Dr Elsa PAROT-SCHINKEL  Cellule de Méthodologie et Biostatistiques  CHU Angers  🕿 02 41 35 58 55  Elparot@chu-angers.fr |
| Biostatistiques | Pr. Christophe BAUFRETON  Service de chirurgie cardiaque  🕿 02 41 35 45 73  chbaufreton@chu-angers.fr |
| Data Manager | Mr Jean-Marie CHRETIEN  Cellule de Méthodologie et Biostatistiques  CHU Angers  🕿 02 41 35 59 76  jmchretien@chu-angers.fr |

## Investigateurs

### Investigateur coordonnateur

Dr Olivier Fouquet, Unité UMR CNRS 6214-INSERM 1083, CCVT, CHU Angers

### Investigateurs associés

Les investigateurs associés et leurs coordonnées sont détaillés dans un document annexe intitulé « Liste des investigateurs associés ».

## Comité scientifique et de pilotage

- Scientifiques associés

Dr Daniel Henrion, responsable unité UMR CNRS 6214-INSERM 1083

Dr Laurent Loufrani, Unité UMR CNRS 6214-INSERM 1083

Dr Frédéric Pinaud, Unité UMR CNRS 6214-INSERM 1083, CCVT, CHU Angers

Equipe de perfusionniste de CCVT, CHU Angers : Emmanuelle Bouquet, Laurence Verron, Antoine Marcesche, Anthony Fribault, Sébastien Girardot.

Emilie Dalmayrac, Ingénieur Attachée de Recherche Clinique, CCVT, CHU Angers

Elise Houssin, Technicienne d’Etudes Cliniques, CCVT, CHU Angers

- Comité scientifique

Dr Daniel Henrion, responsable unité UMR CNRS 6214-INSERM 1083

Pr Christophe Baufreton, PRES LUNAM, Université d’Angers, UPRES EA 3860, CCVT, CHU Angers

# Justification scientifique et description générale de la recherche

## Dénomination et description de la maladie

L’athérosclérose entraîne une obstruction progressive des artères et parfois des complications thrombotiques aiguës. L’infarctus du myocarde (IDM) est une nécrose ischémique systématisée du muscle cardiaque dont l’incidence est de 120 000 cas en France et responsable encore de 10 à 12% de la mortalité annuelle chez l’adulte. La revascularisation coronaire chirurgicale ou pontage coronaire est estimée à 13528 procédures entre le 01/01/2010 et 31/10/2010 selon la base de données élémentaires Epicard de la SFCTCV (Société Française de Chirurgie Thoracique et Vasculaire). Le nombre moyen d’artères vascularisées était de 2.91 par patients. En 2011, selon la même base de données, 58,7% des procédures ont été associées à l’utilisation de 2 greffons artériels thoraciques internes (ATI), 35.4% une seule ATI et 5.8% de greffons saphènes uniquement. L’utilisation des 2 greffons ATI concerne moins de 4% des patients revascularisés aux USA (1). Loop et al. ont démontré la supériorité sur la survie à long terme des ATI gauche sur l’artère interventriculaire antérieure par rapport aux greffons saphènes (2).

En pratique courante, aucun consensus n'a été établi sur le choix de la pompe artérielle à utiliser. Ainsi, chaque centre, en fonction des habitudes, utilise comme pompe de perfusion artérielle en CEC : une pompe à galets et/ou une pompe centrifuge.

## Dénomination et description des dispositifs / stratégies / procédures étudié(e)s

Ce projet porte sur deux types de pompes de perfusion artérielle utilisés en routine en CEC :

- **Pompes à galets dites occlusives** : les plus couramment utilisées, elles fonctionnent sur le principe de l’occlusion plus ou moins complète d’un tube souple en silicone par des galets rotatifs. Le débit est fonction du diamètre du tube (en général 1/2 pouce chez l’adulte), de la circonférence du support et du nombre de tours/minute de la pompe (50-150 rpm). On règle les galets de manière à obtenir une subocclusion qui évite d’écraser les éléments figurés mais qui est suffisante pour propulser la masse sanguine. Les pompes à galets sont indépendantes de la postcharge: elles maintiennent leur débit quelle que soit la pression artérielle.

- **Pompes centrifuges dites non-occlusives** : Elles propulsent le sang par l’action d’une turbine rotative. La différence de pression entre le centre et la périphérie créée par la force centrifuge à l’intérieur du cône accélère le sang qui est éjecté à l’extérieur. Cette pompe traumatise peu les éléments figurés et réduit la stimulation plaquettaire et l’hémolyse, mais elle est sensible à la précharge et à la postcharge car elle n’est pas occlusive: une augmentation des résistances artérielles diminue son débit.

Les dispositifs étudiés sont détaillés dans le chapitre 6.

## Résumé des résultats des essais non cliniques et des essais cliniques disponibles et pertinents au regard de la recherche en soins courants concernée

Les greffons artériels de type artères thoraciques internes (ATI) constituent le meilleur greffon en terme de perméabilité à long terme. Ces artères musculaires ou de résistances constituent une grande part de l’arbre vasculaire. Ces artères de petit calibre contiennent une grande proportion de cellules musculaires lisses par rapport aux artères élastiques (artères de plus gros calibres) et jouent un rôle prépondérant dans la régulation systémique de la pression artérielle, pour une distribution optimale du sang au niveau du tissu. De nombreuses études ont comparé la perméabilité des greffons: 98% à 5 ans, 95% à 10 ans et 88% à 15 ans pour les artères thoraciques internes gauches (9). Dans cette étude Tatoulis et al. (9) ont montré que la perméabilité du greffon dépendait du type de conduit utilisé, de leur distribution et du degré de sténose coronaire d’aval. D’autres greffons artériels sont également utilisés comme les artères radiales. Néanmoins, contrairement aux artères thoraciques internes prélevées in-situ, les artères radiales sont comme les veines saphènes, utilisées comme des autogreffes, soumises à une séquence d’ischémie-reperfusion tissulaire lié à la procédure comprenant la perte du flux pulsatile en l’absence de débit et pression dans la lumière artérielle. Il est intéressant de constater que les artères radiales comme les veines saphènes sont soumise au phénomène d’hyperplasie intimale (10). Les études cliniques comparatives ont démontré l’absence de supériorité des artères radiales sur les veines saphènes en terme de perméabilité postopératoire à distance de l’intervention (10, 11). En ce qui concerne l’utilisation des greffons artériels ou veineux comme autogreffe pour pontage, peu de données ont montré l’impact de la pulsatilité, ou de son absence, sur la structure et la fonction endothéliale.

En 2010, 87.8% des procédures de revascularisation coronaire ont été réalisées sous circulation extra-corporelle (CEC) selon les données Epicard. Les pompes de perfusion artérielle en CEC sont :

- Occlusives : encore largement utilisées, elles sont constituées de 2 galets qui tournent autour d’un tuyau en PVC provoquant un flux par compression du sang

- Non-occlusives : par étirement ou centrifuge, entraînent moins d’hémolyse et sont issues de l’assistance mécanique.

Les pompes à galets produisent un flux pulsé (12) et peuvent être réglées pour renforcer ou non le débit pulsé. La pulsatilité d’un flux dépend plus du gradient d’énergie que du gradient de pression (13). Durant l’opération, l’utilisation d’une pompe centrifuge générant un flux non pulsé est associée à une augmentation de la réponse inflammatoire (activation du complément et des neutrophiles) pour des raisons encore mal élucidées (14). Une étude récente a montré sur un modèle in-vitro que la perte de la pulsatilité dans une artère mésentérique de rat (artère de résistance), indépendamment du flux ou de la pression, génère après 30 minutes une réponse inflammatoire pariétale liée à un stress oxydatif pouvant être supprimé par l’utilisation d’anti-oxydant (tempol) (15) La présence d’une pulsatilité, dans cette étude in vitro, entraîne une diminution du tonus myogénique et une dilatation précoce au flux. En l’absence de pulsatilité, aucune modification de la réactivité pharmacologique n’a été constatée et la pression non pulsée entraîne une production importante des protéines inflammatoires et ROS. Lors d’une CEC pour pontage, la phase opératoire pendant laquelle la pulsatilité peut être abolie correspond à celle du clampage aortique lorsque l’activité cardiaque native a cessé du fait de la cardioplégie. La pulsatilité résiduelle dans le système vasculaire au cours du clampage aortique est donc sous la seule dépendance de la pompe artérielle utilisée. Watarida et al ont montré chez des patients opérés de pontages aorto-coronariens que l’élévation des concentrations circulantes d’endotoxine circulante était corrélée à la durée de clampage en présence d’une CEC non pulsatile (16). Dans une revue de la littérature publiée par Alghamdi (17), le seul essai contrôlé et randomisé jugé de bonne qualité a permis de conclure qu’une perfusion pulsatile (pompe à galets) durant la CEC était associée à une réduction des infarctus myocardiques, de la mortalité et des complications majeures (18). Cependant, les pompes centrifuges délivrant un débit non pulsé, restent largement utilisées.

En conséquence, il nous semble indispensable de pouvoir évaluer l’impact de la pulsatilité sur les greffons artériels afin de déterminer si une pompe centrifuge (non pulsatile) peut être délétère sur la fonction mécanique de ces greffons pouvant expliquer certaines occlusions précoces.

## Justification de la qualification de la recherche en soins courants

L’objectif général de ce projet en soins courants est d’évaluer et de comparer l'impact sur la fonctionnalité endothéliale des ATI de deux types de pompes de perfusion artérielle utilisés en routine en CEC (pompes à galets et pompes centrifuges) :

1. Le service de chirurgie cardiaque du CHU d'Angers a réalisé en 2012, 637 actes de chirurgie cardiaque majeure. Sur la même année, 202 patients ont été opérés de pontages coronaires seuls avec plus de 98% d'ATI utilisée. 92% des procédures ont été réalisées sous CEC. Une dotation de 50 pompes centrifuges par an a été attribuée au service, l'utilisation de ces pompes étant faite de façon aléatoire pour permettre aux cardioperfusionnistes de conserver "une habitude" de la technique. L’utilisation de ces pompes se fait de façon aléatoire autant pour la chirurgie coronaire que pour la chirurgie valvulaire.
2. Les seules investigations spécifiques à l’étude sont les prélèvements sanguins réalisés au bloc ainsi que l’étude des prélèvements tissulaires (segments d’artères thoraciques usuellement considérés comme des déchets opératoires). Il n’y a donc aucun risque et contrainte spécifique pour le patient dans cette étude.
3. Les connaissances actuelles sur l'impact d'une pompe centrifuge ou à galets ne permet pas de pouvoir considérer une technique supérieure à une autre. Aucune n'ayant montrée sa supériorité en termes de bénéfice et de sécurité pour le patient. Cependant, de nombreuses études (14, 16, 17, 18) ont montré que l’utilisation d’une pompe à galets réduisait significativement la réponse inflammatoire générale. Seule l’étude expérimentale réalisée par Pinaud et al. (15) a montré une altération importante de la vasoréactivité artérielle (artères mésentériques de rat) en l’absence d’un flux pulsé.Il n'existe aucune base de données sur l'utilisation des types de pompes utilisée en France, seule une enquête téléphonique auprès de 58 centres de chirurgie cardiaque français (sur 65) a révélé qu'une pompe centrifuge était utilisée dans 34% (n=20) des cas.

## Description de la population à étudier

Les patients de sexe masculin devant avoir une revascularisation chirurgicale avec au moins une ATI seront inclus dans cette étude.

# Hypothèses et objectifs de la recherche

## Hypothèses

L’hypothèse du protocole I.P.A.T.I est que l’utilisation d’une pompe à galets délivrant une pulsatilité à la microcirculation pendant le clampage aortique, aurait un impact plus favorable sur la fonctionnalité endothéliale des ATI que l’utilisation d’une pompe centrifuge car elle n’entraînerait pas d’altération du tonus myogénique de l’artère thoracique interne (hypothèse élaborée à partir des conclusions de l’étude expérimentale de Pinaud et al.)

Si notre hypothèse s’avérait exacte, il nous semblerait justifié de modifier nos habitudes chirurgicales c’est-à-dire l’utilisation systématique d’une pompe à galets au décours d’une chirurgie pour pontages coronaires.

## Objectifs et critères de jugement

Les objectifs seront d’évaluer en fonction du type de pompes utilisé:

- la vasoréactivité de l’artère thoracique interne ;
- le stress oxydative et réaction inflammatoire au niveau de la paroi du greffon ;
- la réaction inflammatoire dans le sang du patient.

Les critères de jugement correspondant sont :

- pourcentage de contraction en fonction de la pression (tonus myogénique) et le pourcentage de dilatation en fonction du débit (vasodilatation) ainsi que les phénomènes de dilatation et contraction en fonction d'ajouts pharmacologiques ;
- mesure ROS (reactive oxygen species), étude histologie, MCP-1 (Monocyte chemotactic protein-1), TNF-α ;
- dosage de la chaîne du complément sC5b-9 et de l’élastase leucocytaire.

# Conception de la recherche

## Justification du nombre prévu de personnes à inclure dans la recherche

Il n’existe pas de données dans la littérature permettant d’effectuer un calcul d’effectif pour cette étude exploratoire de physiopathologie. Le seul article assez proche en termes de méthodologie et d’objectifs (15), porte sur une étude expérimentale sur des artères mésentériques de rats réalisée avec 10 rats par groupe soit une centaine de rats au total.

Un nombre de 40 patients par groupe (groupe « pompe à galets » ou groupe « pompe centrifuge ») est un standard habituellement retenu dans ce type de recherche. Chaque patient aura 2 prélèvements tissulaires : 1 avant le clampage et 1 pendant le clampage juste avant l’anastomose. Chaque prélèvement tissulaire servira à l’étude de la vasoréactivité, biochimie et histologie.

## Description des mesures prises pour réduire et éviter les biais

### Tirage au sort

L’allocation des produits sera réalisée par une randomisation équilibrée. La procédure de randomisation est réalisée par la Cellule de Méthodologie et Biostatistiques du CHU d’Angers. Les normes de sécurité prévues à Angers, pour toute intervention sous CEC prévoient que la pompe soit montée à l’arrivée du patient au bloc opératoire.

Ainsi les patients seront randomisés la veille de l’intervention afin que la préparation du matériel nécessaire à l’intervention prévoie la pompe de perfusion pour la CEC (pompe à galets ou pompe centrifuge) conformément à la randomisation.

### Méthodes de mise en insu

Il s’agit d’une étude en simple aveugle car le chirurgien et les cardioperfusionnistes connaissent obligatoirement la pompe montée sur le circuit. Ainsi, seul le patient est en aveugle en ce qui concerne le choix de la pompe.

Cependant, les prélèvements sanguins et tissulaires étant anonymisés, les ingénieurs de laboratoires réalisant les différentes expérimentations et analyses ne connaîtront pas le type de pompe utilisée donc l’évaluation des critères de jugement de l’étude sera faite en aveugle.

## Description de la méthodologie de la recherche, accompagnée de sa présentation schématique précisant notamment les visites et les examens prévus

### Plan expérimental

Il s’agit d’une étude en soins courants, monocentrique, comparative, prospective, en simple insu, randomisée.

Cette étude portera sur des patients opérés de pontage coronaire, au CHU d’Angers, chez qui des segments d’artère thoracique interne nécessaire aux pontages ainsi que des prélèvements biologiques seront analysés afin d’étudier l’impact physiopathologique du type de pompe artérielle utilisée au décours de la CEC.

### Déroulement de l'étude

#### Recueil de la non-opposition / Inclusion

La pré-sélection des patients se fera en réunion médico-chirurgicale, à partir de leur dossier médical et de leur précédente consultation, selon les différents critères de sélection évaluables à cette date, et sans en avertir le sujet.

Lors de la visite d’inclusion, au moment de la consultation de chirurgie, si le sujet remplit les critères de sélection de l’étude, sa non-opposition est recueillie par l’investigateur après un entretien et la délivrance de la lettre d’information écrite dans un langage compréhensible par le sujet. La lettre d’information est signée au moins en 2 exemplaires par les différentes parties.

#### Suivi des personnes se prêtant à la recherche

A l’occasion de la visite réalisée la veille de l’intervention (J-1), un examen clinique (cf chapitre 7.1.2) sera réalisé afin de vérifier l’absence de contre-indications opératoires récentes (infections, déstabilisation de la maladie coronaire, trouble de l’hémostase, etc..) et de randomiser le patient.

Au cours de l’intervention (J0), les données relatives à l’intervention ainsi que les prélèvements tissulaires et biologiques seront recueillis.


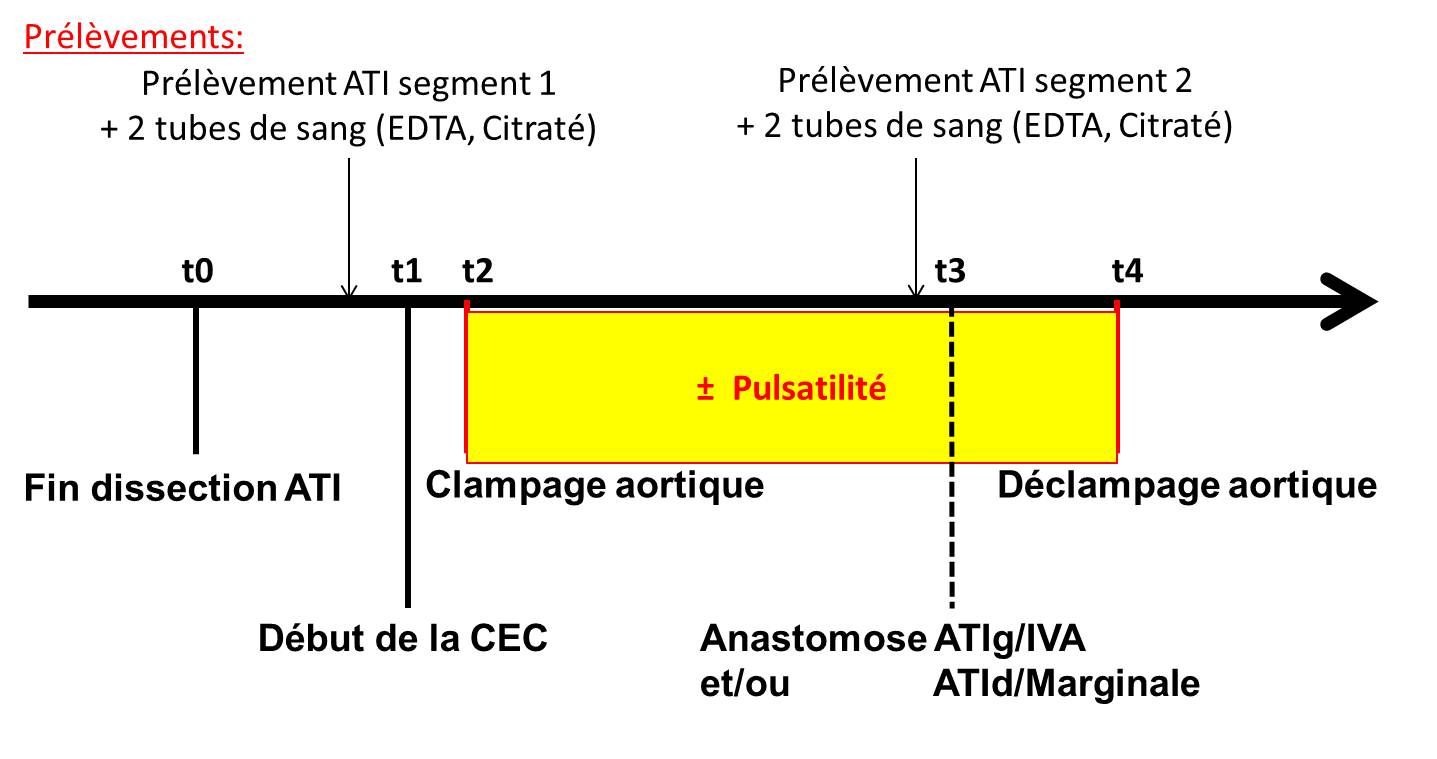


Au quotidien, au cours des pontages coronaires, l’artère thoracique interne (ATI) pédiculée (c’est à dire branchée à sa partie proximale au niveau de l’artère sous-clavière) est prélevée dans sa totalité et fait environ 25 à 30 cm de long. Afin d’adapter la longueur adéquate en fonction du patient, un segment d’environ 5 cm est coupé, systématiquement, dans sa partie distale, ce surplus constituant alors un déchet opératoire.

Ce surplus d’artère constituera la base de l’étude tissulaire des ATI.

Les segments d’ATI seront prélevés en deux temps :

- Juste avant le clampage (artère qui sera alors divisée en 3 parties selon la taille du surplus de greffon: 5 mm pour l’immunohistochimie, 5 mm pour la biochimie et 5 mm pour la vasoréactivité).
- Avant l’anastomose (artère qui sera également divisée en 3 parties selon la taille du surplus de greffon).

Par ailleurs, quatre tubes de sang (2 EDTA, 2 citraté) seront prélevés pendant l'intervention dans le cadre de ce protocole. Les prélèvements auront lieu à juste avant le prélèvement de chaque segment d’ATI:

- 1 tube EDTA (quantité 3 ml), 1 tube citraté (quantité 4 ml) juste avant le prélèvement du segment 1 d'ATI
- 1 tube EDTA, 1 tube citraté juste avant le prélèvement du segment 2 d'ATI

Au total, une quantité de 14 ml de sang sera prélevée dans le cadre de cette étude.

L’étude ne prévoit pas de suivi clinique des patients après l’intervention. En effet, il sera impossible sur un nombre restreint de sujet de déterminer l'impact clinique à distance du choix du type de pompe.

## Durée prévue de participation des personnes et description de la chronologie et de la durée de toutes les périodes de l’essai, y compris le suivi, le cas échéant

Nombre de patients prévus : 80

Durée de la période d’inclusion : 2 ans

Délai entre l’inclusion et la chirurgie : délai habituel pour ce type d’intervention (environ 1 mois)

Durée de participation à l'étude pour un patient: Le temps de la mise sous CEC pendant la chirurgie coronaire. (+/- 1 heure)

Durée totale de l'étude (durée d’inclusion + durée de participation) : 2 ans et 1 mois

## Description des règles d’arrêt définitif ou temporaire

### Arrêt de participation d’une personne à la recherche

Les patients pourront demander à sortir de l’étude à n’importe quel moment et quelle qu’en soit la raison.

L’investigateur pourra interrompre définitivement la participation d’un patient à l’étude pour toute raison qui servirait au mieux les intérêts du patient.

Les critères d’exclusion du patient sont :

- Opposition du patient à l’utilisation de ses données

- Contre-indication à la chirurgie ou annulation de l’intervention prévue

- Longueur du greffon trop petite pouvant compromettre la qualité du pontage

- Contexte hémodynamique et/ou hémorragique précaire rendant le temps des prélèvements non adapté.

Les patients sortis d’étude avant la randomisation seront remplacés (inclusion et randomisation de 80 patients).

La sortie d'étude d'un patient ne changera en rien sa prise en charge habituelle par rapport à sa maladie.

En cas de sortie prématurée sans opposition du patient à l’utilisation de ses données, l’investigateur doit en documenter les raisons de façon aussi complète que possible.

Les données recueillies pour les sujets sortis prématurément de d’étude seront exploitées au moment des analyses sauf en cas d’opposition du patient à l’utilisation de ses données.

### Arrêt d’une partie ou de la totalité de la recherche

Des événements imprévus, au vu desquels les objectifs de l'étude ou du programme clinique ne seront vraisemblablement pas atteints, peuvent amener le gestionnaire à interrompre prématurément l’étude.

Le CHU d’Angers se réserve le droit d'interrompre l’étude, à tout moment, s'il s'avère que les objectifs d’inclusion ne sont pas atteints.

En cas d’arrêt prématuré de l’étude, l’information sera transmise par le gestionnaire dans un délai de 15 jours au CPP. En cas d’arrêt prématuré de l’étude, les investigateurs doivent en informer rapidement les patients participant à l’étude.

# Sélection et exclusion des personnes de la recherche

## Critères d’inclusion des personnes qui se prêtent à la recherche

> 18 ans

Homme

Chirurgie élective pour pontage coronaire utilisant au moins une artère thoracique interne

Ces patients sont volontaires et ne se sont pas opposés à l’utilisation de leurs données

## Critères de non inclusion des personnes qui se prêtent à la recherche

Femme (en raison de l'impact hormonal (œstrogène) sur la vasoréactivité artérielle

Chirurgie combinée

Chirurgie en urgence

Participation à un autre protocole

Opposition à l’utilisation des données

## Modalités de recrutement

Les patients ayant une intervention programmée dans le service de Chirurgie Cardiaque dans le cadre de leur pathologie se verront proposé de participer à l'étude.

# Prise en charge et traitements administrés aux personnes qui se prêtent à la recherche

## Description des dispositifs / stratégies / procédures étudié(e)s

En pratique courante, le choix de la pompe est aléatoire. Les cardioperfusionnistes utilisent à leur convenance des pompes centrifuges ou à galets afin d’assurer une « habitude d’utilisation ». Le circuit de CEC sera identique quelque soit le type de pompe utilisée : circuit Sorin® Référence AB0395.

### Dispositif / stratégie / procédure A

Le dispositif A étudié est la pompe à galets de marque Maquet dont le mécanisme est constitué d'un rotor avec 2 galets comprimant un tuyau en silicone.

### Dispositif / stratégie / procédure B

Le dispositif B étudié est la pompe centrifuge de marque Sorin dont le mécanisme est constitué d'un aimant entraînant l'étirement du sang, cette technique étant issue de l'assistance mécanique

## Autres traitements, dispositifs, stratégies associé(e) s

Il n’y a aucune modification de la prise en charge des patients.

# Critères d’évaluation

## Critères d’évaluation non spécifiques à l’étude

### Visite d’inclusion

Les données cliniques du patient concerneront : l’âge, le sexe, poids, taille, dyslipidémie, hypertension artérielle, tabac, diabète de type 1 et 2, antécédents familiaux cardio-vasculaires, athérosclérose des artères sous-clavières, antiagrégants, maladie inflammatoire associée.

### Veille de l’intervention

Les données de l’examen clinique pré-opératoire seront recueillies ainsi que ainsi que, le cas échéant, les critères d’exclusion (opposition, contre-indication à l’intervention, …).

Pour les données biologiques pré-opératoires: CRP, leucocytes

### Intervention

Les données dites chirurgicales concerneront : le type de pompe utilisée, le type de circuit utilisé, le niveau d’anticoagulation (mesure de l’ACT [activated clotting time] cible sur Hepcon® HMS au bloc opératoire et dose d’héparine injectée).

## Critères d’évaluation spécifiques à l’étude

Il s’agit des critères d’évaluation issus des résultats de l’analyse biologique sanguine et tissulaire. Aucune autre procédure supplémentaire de surveillance ne sera proposée dans le cadre de cette étude.

### Analyses tissulaires

Les segments d’artère thoracique nécessaires à l’étude biochimiques, immunohistochimiques seront congelés grâce à de l’azote liquide (-80°C) dans l’enceinte du bloc opératoire, dans une salle dédiée, par un technicien de laboratoire. Les segments pour étude de la vasoréactivité sont conservés frais et acheminés avec les autres prélèvements au laboratoire UMR CNRS 6214-INSERM 1083 par le technicien.

Les analyses réalisées à partir de ces prélèvements tissulaires seront :

1. Analyse de la vasoréactivité artérielle : pourcentage de dilatation et de contraction en fonction de la pulsatilité et pharmacologie. Les artères ainsi isolées, à l’état frais, seront montées sur un artériographe et perfusées avec un sérum salé physiologique sans cellules circulantes.

- Evaluation du tonus myogénique : mesure du diamètre passif de l’artère lorsque celle-ci est soumise à différents régimes de pression (10, 25, 50, 75, 100, 125 et 150 mmHg) en fonction du temps.
- Evaluation de la vasodilatation (dilatation flux dépendant): mesure du diamètre de l’artère en fonction du débit circulant dans l’artère qui est augmenté étape par étape de 0 à 100 μl/min en fonction du temps.
- Etude de la vasoréactivité en fonction d’ajout d’agents pharmacologique : antioxydant (TEMPOL)

1. Analyse du stress oxydatif pariétal : mesure ROS (reactive oxygen species)
2. Analyse histologie
3. Analyse immunohistochimique : MCP-1 (Monocyte chemotactic protein-1), TNF-α

### Analyses sanguines

Quatre tubes de sang seront prélevés juste avant le prélèvement de chaque segment de greffons :

- 2 tubes EDTA (quantité de 3 ml de sang/tube) pour l’étude de la chaîne du complément : sC5b-9

- 2 tubes citratés (quantité de 4 ml de sang/tube) pour l'étude de l’élastase leucocytaire

# Evaluation de la sécurité

S'agissant d'une étude en soins courants, étant donné que les actes sont réalisés conformément aux pratiques habituelles, la déclaration des effets indésirables liés à la prise en charge relève du circuit habituel des vigilances du centre hospitalier où se déroule la recherche : pharmacovigilance si l'effet implique un médicament, matériovigilance pour un dispositif médical, gestion des risques, etc.

## Description des paramètres d’évaluation de la sécurité

En ce qui concerne les examens spécifiques à la surveillance dans le cadre du protocole d’étude, il n’y a pas lieu de prévoir d’événement indésirable secondaire.

Les tubes de sang seront prélevés sur des perfusions préalablement posées pour d’autres analyses, les prélèvements étant effectués au bloc opératoire sous anesthésie générale.

## Comité de Surveillance

Compte tenu de la nature de l’étude et l’absence d'effets indésirables attendus liés aux investigations spécifiques à l’étude, il n’est pas prévu de comité de surveillance.

# Résumé des bénéfices, le cas échéant, et des risques prévisibles et connus pour les personne se prêtant à la recherche

## Bénéfices

### Bénéfice individuel

Il n’y a pas de bénéfice individuel attendu sachant qu’il s’agit d’une étude en soins courants.

### Bénéfice collectif

Cette étude permettra de mieux comprendre les mécanismes de détérioration des greffons mammaires pouvant entraîner des résurgences d’angine de poitrine, d’infarctus. S’il s’avérait que l’absence de débit pulsé pendant la CEC entraînait une altération importante de la paroi du greffon, l’utilisation systématique d’une pompe à galets pour les pontages coronaires devra être envisagée.

## Risques

Aucun risque individuel ou collectif n’est à prévoir dans cette étude en soins courants.

Les prélèvements tissulaires correspondent, en pratique, à des déchets opératoires.

Les tubes de sang seront prélevés sur des perfusions préalablement posées pour d’autres analyses, les prélèvements étant effectués au bloc opératoire sous anesthésie générale.

## Balance bénéfices / risques

Les risques et les contraintes prévisibles pour les patients participant à l’étude, décrits dans les paragraphes ci-dessus sont minimes au regard des bénéfices attendus.

# Traitement des données et gestion des données

## Cahier d’observation

Le cahier d’observation est en cours de réalisation et se composera des données détaillées ci-dessus.

Toutes les informations requises par le protocole doivent être consignées sur les cahiers d’observation et une explication doit être apportée pour chaque donnée manquante. Les données devront être recueillies au fur et à mesure qu'elles sont obtenues, et transcrites dans ces cahiers de façon nette et lisible.

Les données erronées relevées sur les cahiers d'observation seront clairement barrées et les nouvelles données seront copiées, à côté de l'information barrée, accompagnées des initiales, de la date et éventuellement d’une justification par l’investigateur ou la personne autorisée à faire cette correction.

## Saisie des données

La saisie des données sera réalisée par le Docteur Olivier Fouquet, chirurgien cardiaque, CCVT sur des cahiers de recueil des données anonymisés*.*

L'analyse des données sera réalisée par les docteurs Daniel Henrion, Laurent Loufrani et Olivier Fouquet.

## Archivage

Les documents suivants seront archivés à la cellule promotion du CHU d'Angers et dans les locaux du service de chirurgie cardiaque du CHU d’Angers jusqu’à la fin de la période d’utilité pratique selon la réglementation en vigueur.

Ces documents sont :

- Protocole et annexes, amendements éventuels,
- Lettres d’information signées (non-opposition)
- Données individuelles (copies authentifiées de données brutes)
- Documents relatifs aux produits à l'étude
- Analyses statistiques
- Rapport final de l’étude ou résumé du rapport final

A l’issue de la période d’utilité pratique, l’ensemble des documents à archiver sera placé sous la responsabilité du gestionnaire et des investigateurs principaux pendant 15 ans après la fin de l’étude conformément aux pratiques institutionnelles.

Aucune destruction ne pourra être effectuée sans l’accord du gestionnaire. Au terme des 15 ans, le gestionnaire sera consulté pour destruction. Toutes les données, tous les documents et rapports pourront faire l’objet d’audit ou d’inspection.

# Contrôle et assurance de la qualité

L’investigateur principal s’assurera de la bonne réalisation de l’étude, du recueil des données, de leur documentation, enregistrement et rapport, en accord avec les Procédures Opératoires Standards mises en application au sein du CHU d’Angers et conformément aux Bonnes Pratiques Cliniques ainsi qu’aux dispositions législatives et réglementaires en vigueur.

# Statistiques

L’analyse statistique sera réalisée au sein de l’unité UMR CNRS 6214-INSERM 1083 (Dr Daniel Henrion) et du service de chirurgie cardiaque (Pr Christophe Baufreton).

Logiciels utilisés : SPSS V15 (Chicago, IL, USA)

## Description des méthodes statistiques prévues, y compris du calendrier des analyses intermédiaires prévues

Le diagramme de sélection des participants à l’étude sera réalisé ainsi qu’une analyse descriptive des caractéristiques des participants.

Pour les variables qualitatives, les résultats seront rapportés en effectifs et pourcentages. Pour les variables quantitatives, les résultats seront rapportés en moyenne et écart-type en cas de distribution normale et en médiane avec 25° et 75° centile en l'absence de distribution gaussienne de la variable considérée.

Pour les analyses bivariées, l’utilisation de tests paramétriques ou non-paramétriques, sera fonction des effectifs et/ou de la distribution des variables. Le seuil de significativité est fixé à 0,05 et tous les tests seront bilatéraux.

Le test statistique utilisé sera le test de Chi-deux de Pearson (ou le test exact de Fisher) pour les variables qualitatives. Pour les variables quantitatives, les tests statistiques utilisés seront le test t de Student (ou le test non paramétrique de Mann-Whitney) pour la comparaison de 2 groupes et une ANOVA (ou le test non paramétrique de Kruskall Wallis) pour la comparaison de 3 groupes ou plus.

## Degré de signification statistique prévu

Le seuil de significativité est fixé à 0,05 et tous les tests seront bilatéraux.

## Critères statistiques d’arrêt de la recherche

Non applicable.

## Méthode de prise en compte des données manquantes, inutilisées ou non valides

Aucune méthode d’imputation ne sera utilisée dans le cas où des données seraient manquantes.

## Gestion des modifications apportées au plan d’analyse de la stratégie initiale

L’analyse statistique sera réalisée selon un plan d’analyse pré-établi.

## Choix des personnes à inclure dans les analyses

Toutes les personnes pour lesquelles les critères d'évaluation seront disponibles seront prises en compte dans les analyses.

# Droit d’accès aux données et documents source

## Accès aux données

Les données médicales de chaque patient ne seront transmises qu’à l’organisme de rattachement de la personne responsable de la recherche ou toute personne dûment habilitée par celui-ci dans les conditions garantissant leur confidentialité.

## Documents source

Le cas échéant, l’organisme de rattachement de la personne responsable pourra demander un accès direct au dossier médical pour vérification des procédures et/ou des données de la recherche, sans violer la confidentialité et dans les limites autorisées par les lois et régulations.

# Considérations éthiques et réglementaires

## Comité de Protection des Personnes et Autorité compétente

Le dossier de l’étude (notamment le protocole, la liste des investigateurs associés et la lettre d'information de l'étude) sera soumis pour avis au Comité de Protection des Personnes.

Dès la première inclusion, le gestionnaire doit informer sans délai le CPP de la date effective de démarrage de l’étude

La date de fin d’étude sera transmise par le gestionnaire au CPP dans un délai de 90 jours. La date de fin de la recherche correspond au terme de la participation de la dernière personne qui se prête à la recherche, ou le cas échéant, au terme défini dans le protocole.

## Modifications substantielles

En cas de modification substantielle apportée au dossier de l’étude par l’investigateur, elle sera approuvée par le gestionnaire. Ce dernier devra obtenir préalablement à sa mise en œuvre un avis favorable du CPP.

## CNIL

Conformément à la Loi n°78-17 du 6 janvier 1978 relative à l’informatique, aux fichiers et aux libertés modifiée par Loi du 1 août 1994 relative au traitement de données nominatives ayant pour fin la recherche dans le domaine de la santé et la Loi du 6 août 2004 relative à la protection des personnes physiques à l’égard du traitement des données à caractère personnel, s’agissant d’un traitement de données strictement monocentrique, seule une autorisation concernant la mise en œuvre du traitement des données nécessaire à la réalisation de l’étude sera demandée à la Commission Nationale Informatique et Libertés (CNIL).

## Confidentialité des données

Le responsable de la recherche et les personnes ayant un accès direct aux données prendront toutes les précautions nécessaires en vue d’assurer la confidentialité des informations relatives aux personnes qui s’y prêtent et notamment en ce qui concerne leur identité. Ces personnes sont soumises au secret professionnel (selon les conditions définies par les articles 226-13 et 226-14 du code pénal). Dans le cas présent les prélèvements de tissu et sanguin seront transmis de façon anonyme au responsable de la recherche sans information permettant l’identification du donneur.

## Assurance

Dans la mesure où la recherche est bien qualifiée de Recherche en soins courants par le CPP sollicité, ce qui signifie l’absence de risque supplémentaire liée à l’étude, l’assurance sera celle de l’établissement responsable des soins (article L. 1142-2).

Le CHU d’Angers a souscrit auprès de la SHAM un contrat d’assurance (numéro 127049) de responsabilité civile envers les patients, afin de couvrir les obligations mises à sa charge dans le cadre des soins.

# Faisabilité de l'étude

Le service de chirurgie cardiaque du CHU d’Angers a réalisé 637 actes de chirurgies majeures en 2012, et plus de 10 000 CEC en 20 ans avec une reconnaissance nationale des études menées sur la CEC.

En 2012, en dehors des gestes valvulaires associés, 202 patients ont eu des pontages coronaires ; le nombre d’anastomose par patient étant de 2,5/patients. Dans plus de 90% des procédures réalisées, une ATI a été utilisé et 92% des interventions seront réalisées sous CEC.

# Règles relatives à la publication

Les communications et rapports scientifiques correspondant à cette étude seront réalisés sous la responsabilité du responsable de la recherche avec l’accord des chercheurs associés. Les coauteurs du rapport et des publications seront les cliniciens impliqués, au prorata de leur contribution à l’étude, ainsi que le biostatisticien et les chercheurs associés.

Les règles de publications suivront les recommandations internationales (N Engl J Med, 1997; 336 :309-315).

L’étude peut être enregistrée sur un site web en libre accès (Clinical trial) avant le recueil des données du 1er patient.

# Références à la littérature scientifique et aux données pertinentes servant de référence pour la recherche

1. Sabik JF. Understanding Saphenous Vein Graft Patency. Circulation. 2011Jul.18;124(3):273–5.

2. Loop FD, Lytle BW, Cosgrove DM, Stewart RW, Goormastic M, Williams GW, et al. Influence of the internal-mammary-artery graft on 10-year survival and other cardiac events. N Engl J Med. 1986Jan.2;314(1):1–6.

3. Sharma GV, Deupree RH, Khuri SF, Parisi AF, Luchi RJ, Scott SM. Coronary bypass surgery improves survival in high-risk unstable angina. Results of a Veterans Administration Cooperative study with an 8-year follow-up. Veterans Administration Unstable Angina Cooperative Study Group. Circulation. 1991Nov.;84(5 Suppl):III260–7.

4. Thatte H, Khuri S. The coronary artery bypass conduit: I. Intraoperative endothelial injury and its implication on graft patency. Ann Thorac Surg. 2001;72(6):S2245–52.

5. Lopes RD, Mehta RH, Hafley GE, Williams JB, Mack MJ, Peterson ED, et al. Relationship Between Vein Graft Failure and Subsequent Clinical Outcomes After Coronary Artery Bypass Surgery. Circulation. 2012Feb.13;125(6):749–56.

6. Rousou L, Taylor K, Lu X, Healey N, Crittenden M, Khuri S, et al. Saphenous vein conduits harvested by endoscopic technique exhibit structural and functional damage. Ann Thorac Surg. 2009;87(1):62–70.

7. Sun Q, Kawamura T, Masutani K, Peng X, Sun Q, Stolz DB, et al. Oral intake of hydrogen-rich water inhibits intimal hyperplasia in arterialized vein grafts in rats. Cardiovasc Res. 2012Apr.1;94(1):144–53.

8. Mehta RH, Ferguson TB, Lopes RD, Hafley GE, Mack MJ, Kouchoukos NT, et al. Saphenous Vein Grafts With Multiple Versus Single Distal Targets in Patients Undergoing Coronary Artery Bypass Surgery: One-Year Graft Failure and Five-Year Outcomes From the Project of Ex-Vivo Vein Graft Engineering via Transfection (PREVENT) IV Trial. Circulation. 2011Jul.18;124(3):280–8.

9. Tatoulis James, FRACS, Buxton F. Brian, FRACS, and Fuller John A, FRACP. Patencies of 2,127 Arterial Conduits over 15 Years. Ann Thorac Surg 2004;77:93-101.

10. Buxton BF, Hayward PAR, Newcomb AE, Moten S, Seevanayagam S, Gordon I. Choice of conduits for coronary artery bypass grafting: craft or science? European Journal of Cardio-Thoracic Surgery. 2009Apr.1;35(4):658–70.

11. Sun JCJ, Teoh KHT, Lamy A, Sheth T, Ellins ML, Jung H, et al. Randomized trial of aspirin and clopidogrel versus aspirin alone for the prevention of coronary artery bypass graft occlusion: the Preoperative Aspirin and Postoperative Antiplatelets in Coronary Artery Bypass Grafting study. Am Heart J. 2010Dec.1;160(6):1178–84.

12. James SA, Peters J, Maresca L, Kalush SL, Trigueros EA. The roller pump does produce pulsatile flow. J Extra Corpor Technol. 1987Oct.27;19:376–83.

13. Driessen JJ, Dhaese H, Fransen G, Verrelst P, Rondelez L, Gevaert L, et al. Pulsatile compared with nonpulsatile perfusion using a centrifugal pump for cardiopulmonary bypass during coronary artery bypass grafting. Effects on systemic haemodynamics, oxygenation, and inflammatory response parameters. Perfusion. 1995;10(1):3–12.

14. Baufreton C, Intrator L, Jansen PG, Velthuis te H, Le Besnerais P, Vonk A, et al. Inflammatory response to cardiopulmonary bypass using roller or centrifugal pumps. Ann Thorac Surg. 1999Apr.1;67(4):972–7.

15. Pinaud F, Loufrani L, Toutain B, Lambert D, Vandekerckhove L, Henrion D, et al. In vitro protection of vascular function from oxidative stress and inflammation by pulsatility in resistance arteries. J Thorac Cardiovasc Surg. 2011Nov.;142(5):1254–62.

16. Watarida S, Mori A, Onoe M, Tabata R, Shiraishi S, Sugita T, et al. A clinical study on the effects of pulsatile cardiopulmonary bypass on the blood endotoxin levels. J Thorac Cardiovasc Surg. 1994Oct.;108(4):620–5.

17. Alghamdi AA, Latter DA. Pulsatile versus nonpulsatile cardiopulmonary bypass flow: A evidence-based approach. J Card Surg. 2006;21:347-354.

18. Murkin JM, Martzke JS, Buchan AM, Bentley C, Wong CJ. A randomized study of the influence of perfusion technique and ph management strategy in 316 patients undergoing coronary artery bypass surgery. Neurologic and cognitive outcomes. The Journal of thoracic and cardiovascular surgery. 1995;110:349-362.

# Liste des annexes

Annexe  : Listing investigateurs

**ANNEXE  :** Listing investigateurs

Docteur Olivier FOUQUET, MD

Docteur en Médecine, Praticien Hospitalier, Chirurgie Cardiaque

Chirurgie Cardiaque Vasculaire et Thoracique, CHU Angers

4 rue Larrey 49933 Angers Cedex 9

[olfouquet@chu-angers.fr](mailto:olfouquet@chu-angers.fr)

02 41 35 45 73

UMR CNRS 6214-INSERM 1083: "Biologie neurovasculaire: physiopathologie de la microcirculation, rôle du stress oxydant", Faculté de Médecine d'Angers, Rue Haute de Reculée 49045 Angers

N° ADELI: 491054433

Professeur Christophe BAUFRETON, MD, PhD

Docteur en Médecine et docteur en Sciences

Titulaire d'une Habilitation à Diriger la Recherche

Professeur de chirurgie cardiaque, Praticien Hospitalier

Chirurgie Cardiaque Vasculaire et Thoracique, CHU Angers

4 rue Larrey 49933 Angers Cedex 9

[chbaufreton@chu-angers.fr](mailto:chbaufreton@chu-angers.fr)

02 41 35 45 73

PRES LUNAM, Université d'Angers, UPRES EA 3860 "Cardioprotection, Remodelage, Thrombose"

N° ADELI: 491036695

Docteur Frédéric PINAUD, MD, PhD

Docteur en Médecine et docteur en Sciences, Praticien Hospitalier, Chirurgie Cardiaque

Chirurgie Cardiaque Vasculaire et Thoracique, CHU Angers

4 rue Larrey 49933 Angers Cedex 9

[frpinaud@chu-angers.fr](mailto:frpinaud@chu-angers.fr)

02 41 35 45 73

UMR CNRS 6214-INSERM 1083: "Biologie neurovasculaire: physiopathologie de la microcirculation, rôle du stress oxydant", Faculté de Médecine d'Angers, Rue Haute de Reculée 49045 Angers

N° ADELI: 491047569

Docteur Jean Patrice BINUANI, MD, PhD

Docteur en Médecine, Praticien Hospitalier, Chirurgie Cardiaque

Chirurgie Cardiaque Vasculaire et Thoracique, CHU Angers

4 rue Larrey 49933 Angers Cedex 9

[frpinaud@chu-angers.fr](mailto:frpinaud@chu-angers.fr)

02 41 35 45 73

UMR CNRS 6214-INSERM 1083: "Biologie neurovasculaire: physiopathologie de la microcirculation, rôle du stress oxydant", Faculté de Médecine d'Angers, Rue Haute de Reculée 49045 Angers

N° ADELI: 49 40 4249 5

Professeur Jean-Louis DEBRUX, MD, PhD

Docteur en Médecine et docteur en Sciences

Professeur de chirurgie cardiaque, Praticien Hospitalier

Chirurgie Cardiaque Vasculaire et Thoracique, CHU Angers

4 rue Larrey 49933 Angers Cedex 9

[chbaufreton@chu-angers.fr](mailto:chbaufreton@chu-angers.fr)

02 41 35 45 73

PRES LUNAM, Université d'Angers, UPRES EA 3860 "Cardioprotection, Remodelage, Thrombose"

N° ordre: 49/02984

Docteur Sara FILIPPINI, MD

Docteur en Médecine, Praticien Hospitalier, Chirurgie Cardiaque

Chirurgie Cardiaque Vasculaire et Thoracique, CHU Angers

4 rue Larrey 49933 Angers Cedex 9

[sara.filippini@chu-angers.fr](mailto:sara.filippini@chu-angers.fr)

02 41 35 45 73

N° ordre: 49/6236
