# Supplementary material for: Cardiopulmonary bypass and internal thoracic artery: Can roller or centrifugal pumps change vascular reactivity of the graft? The IPITA study: A randomized controlled clinical trial
Source: PLoS One. 2020 Jul 9;15(7):e0235604. doi: 10.1371/journal.pone.0235604 (PMC7347139; doi:10.1371/journal.pone.0235604)
Supplement: S4 Appendix — (DOC) [file pone.0235604.s007.doc]

**IPATI : LETTRE D’INFORMATION**

**Analyse de l'Impact de la pulsatilité sur la vasoréactivité artérielle et la réponse inflammatoire des Artères Thoraciques Internes_ IPATI**

**Gestionnaire**

*CHU d'Angers*

*4, rue Larrey*

*49933 Angers cedex 9*

**Investigateur coordonnateur**

Nom : Dr FOUQUET Olivier

Service: Chirurgie Cardiaque

Téléphone 02 41 35 34 78

Fax : 02 41 35 52 80

Madame, Mademoiselle, Monsieur,

Vous avez été invité(e) à participer à une étude appelée IPATI visant à évaluer des soins courants.

Bien que cette étude ne modifie en rien votre prise en charge et suivi habituel, vous trouverez ci-après des informations concernant votre participation à cette recherche.

1. **OBJECTIF DE L’ETUDE**

Vous allez être opéré(e) du cœur pour des pontages aorto-coronarien.

Le pontage consiste à réaliser un « pont » entre deux artères permettant « d’enjamber » la zone rétrécie de votre artère coronaire. Le « pont », appelé **greffon**, est constitué d’une artère (situé au niveau du thorax) ou d’une veine (prélevée sur la jambe). L’artère qui sera prélevée pour votre pontage est appelé « **Artère Thoracique Interne**».

Le pontage coronarien est une opération à cœur ouvert. Pendant un moment, les gros vaisseaux qui arrivent au cœur et qui en partent sont déconnectés et branchés sur une machine qui assure temporairement la fonction cardiaque. Le cœur est alors remplacé par une **pompe**, ce qui permet au chirurgien de travailler sur un cœur immobile. Lorsque le cœur se contracte et éjecte le sang dans l’aorte, celui-ci génère une énergie qui va se propager dans l’ensemble des artères. Cette énergie, reflet des contractions du cœur, est à l’origine du pouls cardiaque, on parle alors de pulsatilité.

Il existe actuellement deux types de pompes :

- Pompe à galets : elles produisent une pulsatilité comme le pouls cardiaque,
- Pompe centrifuge : elles ne produisent aucune pulsatilité c’est-à-dire qu’il n’y a aucun pouls.

L'hypothèse, motivant la réalisation d'une telle étude, est que le maintien de la pulsatilité pendant toute la chirurgie, permettrait de moins abîmer la paroi de l'artère qui sera greffée et donc d'améliorer la conservation du greffon artériel à long terme.

1. **DEROULEMENT DE L’ETUDE**

L'étude se déroulera durant votre hospitalisation (les prélèvements ayant lieu au moment de l’opération) sur l'avis de l'un des médecins associés qui participe à l'étude.

En pratique courante (hors protocole), des morceaux d’artères qui serviront à réaliser le pontage, sont coupés afin d’obtenir la longueur adéquate du greffon. Dans le cadre de cette étude, ces morceaux d’**artères**, habituellement non conservés, seront récupérés. 4 tubes de **sang** supplémentaires seront également prélevés au décours de l’opération afin de doser les **facteurs inflammatoires** susceptibles d'influencer la conservation de la paroi de l'artère. Les reliquats éventuels de prélèvements, sanguins et tissulaires, seront détruits après analyse.

Le choix de la pompe sera aléatoire, une randomisation sera effectuée avant votre opération.

Dans tous les cas, le transfert des données vous concernant se fera en toute confidentialité sous forme codée et votre identité ne sera jamais révélée.

En cas d’arrêt prématuré de la recherche, sauf opposition écrite de votre part, nous effectuerons un traitement informatique de vos données personnelles recueillies préalablement à l’arrêt de la recherche.

1. **PARTICIPATION VOLONTAIRE**

**Votre participation à cette étude est entièrement libre et volontaire.**

Votre participation à cette étude est totalement volontaire et vous êtes libre de refuser de participer à l’étude ou de l’interrompre à tout moment sans avoir à vous justifier et sans aucun préjudice quant à la qualité de votre prise en charge médicale.

1. **CONFIDENTIALITE ET UTILISATION DES DONNEES MEDICALES**

Votre participation à cette étude et les données recueillies vous concernant resteront strictement confidentielles. Toutefois, les Autorités de Santé ainsi que les personnes mandatées par le gestionnaire auront un accès direct à votre dossier médical afin de vérifier que l’étude est effectuée en conformité avec la législation en vigueur et les réglementations des Autorités de Santé.

Les données enregistrées au cours de cette étude seront anonymysées et feront l’objet d’un traitement informatisé. Votre droit d’accès et de rectification, prévu par la loi relative à l’informatique, aux fichiers et aux libertés, pourra s’exercer dans les conditions prévues par la réglementation à tout moment auprès des responsables de l’étude. Vous pourrez exercer ce droit directement ou par l’intermédiaire du médecin de votre choix.

1. **PROTECTION DES PERSONNES**

Cette étude sera menée conformément à la loi n°2004-806 du 9 août 2004 ainsi qu’aux textes réglementaires relatifs à la protection des personnes se prêtant à des recherches en soins courants.

La participation à cette étude nécessite que vous soyez affilié(e) ou bénéficiaire d’un régime de sécurité sociale.

1. **AVIS FAVORABLE DU CPP**

Conformément à la loi n°2004-806 du 9 août 2004 relative à la politique de santé publique, le Comité de Protection des Personnes Angers Ouest II a étudié ce projet de recherche en soin courant et a émis un avis favorable à sa réalisation le 16 décembre 2014.

# Toutes les informations que vous souhaiterez obtenir ultérieurement concernant ce programme, ou votre participation vous seront communiquées dans la mesure du possible par le médecin responsable de la recherche, le Dr Olivier Fouquet (🕿 02.41.35.78.96) ou toute personne désignée, par lui-même, pour le représenter. Toute nouvelle information disponible au cours de ce programme et pouvant éventuellement modifier votre décision de participation vous sera rapportée. Si vous l'acceptez, le Dr Olivier Fouquet informera votre médecin traitant (Dr …………………..) de votre participation à ce programme.

# Votre consentement ne dégage ni le gestionnaire, ni les médecins associés à la recherche de leurs responsabilités. Tous les coûts inhérents à ce programme seront à la charge du gestionnaire.

**Pour toute question relative à ce programme, vous pouvez contacter le Dr Olivier Fouquet (🕿 02.41.35.78.96)**

***Medecin associé à/responsable de la recherche***

Nom ……………………………………………………………………………….

Service ……………………………………………………………………………

Certifie que le patient (nom, prénom), ……………………………………………ne s'oppose pas à la participation à l'étude.

Fait à ………………………..……… le ………………….…………………..

SIGNATURE :
